# Phased diploid genome assemblies and pan-genomes provide insights into the genetic history of apple domestication

**DOI:** 10.1038/s41588-020-00723-9

**Published:** 2020-11-02

**Authors:** Xuepeng Sun, Chen Jiao, Heidi Schwaninger, C. Thomas Chao, Yumin Ma, Naibin Duan, Awais Khan, Seunghyun Ban, Kenong Xu, Lailiang Cheng, Gan-Yuan Zhong, Zhangjun Fei

**Affiliations:** 1 Boyce Thompson Institute, Ithaca, NY USA; 2 US Department of Agriculture, Agricultural Research Service, Plant Genetic Resources Unit, Geneva, NY USA; 3 Shandong Centre of Crop Germplasm Resources, Shandong Academy of Agricultural Sciences, Jinan, Shandong China; 4 Plant Pathology and Plant-Microbe Biology Section, School of Integrative Plant Science, Cornell University, Geneva, NY USA; 5 Horticulture Section, School of Integrative Plant Science, New York State Agricultural Experiment Station, Cornell University, Geneva, NY USA; 6 Horticulture Section, School of Integrative Plant Science, Cornell University, Ithaca, NY USA; 7 US Department of Agriculture, Agricultural Research Service, Robert W. Holley Center for Agriculture and Health, Ithaca, NY USA

**Keywords:** Plant genetics, Genomics, Population genetics

## Abstract

Domestication of the apple was mainly driven by interspecific hybridization. In the present study, we report the haplotype-resolved genomes of the cultivated apple (*Malus domestica* cv. Gala) and its two major wild progenitors, *M. sieversii* and *M. sylvestris*. Substantial variations are identified between the two haplotypes of each genome. Inference of genome ancestry identifies ~23% of the Gala genome as of hybrid origin. Deep sequencing of 91 accessions identifies selective sweeps in cultivated apples that originated from either of the two progenitors and are associated with important domestication traits. Construction and analyses of apple pan-genomes uncover thousands of new genes, with hundreds of them being selected from one of the progenitors and largely fixed in cultivated apples, revealing that introgression of new genes/alleles is a hallmark of apple domestication through hybridization. Finally, transcriptome profiles of Gala fruits at 13 developmental stages unravel ~19% of genes displaying allele-specific expression, including many associated with fruit quality.

## Abstract (web summary)

Phased diploid genomes of the cultivated apple *Malus domestica* cv. Gala and its two major wild progenitors *M. sieversii* and *M. sylvestris*, as well as pan-genome analyses, provide insights into the genetic history of apple domestication.

## Main

Crop domestication has played a crucial role in human population expansion and civilization. Today, humans rely heavily on a number of crops that were domesticated thousands of years ago^1^. Genetic improvement of key crops for enhanced traits has been empowered by technical innovations^2,3^, but hindered by the narrow genetic diversity of the domesticated crops. Crop wild relatives represent an important source of genetic material for breeding, and genes underlying desirable traits in these wild relatives are often exploited to improve domesticated germplasm^4,5^. Despite its importance, genomic information for crop wild relatives is scarce^6^.

Most crop plants have complex genomes characterized by large size, high heterozygosity level and polyploidy^7^. Such complexity challenges plant genome assembly, for which additional efforts on reference selection are often required to achieve a good-quality genome, and in many cases a homozygous line with lower ploidy level is favored^8,9^. However, many plants are open pollinated in nature, whereby heterozygous genomic regions can be major contributors to phenotypic variations^10^. Hence, direct sequencing of naturally heterozygous lines can provide deep views on their genetic complexity^11^. On the other hand, plants tend to be genetically structured, and a single reference genome can by no means represent a whole population. Therefore, generation of a sophisticated representation of population diversity in addition to a linear reference genome is of great interest. Variants of such representation, including gene-based^12,13^ or sequence-based^14,15^ pan-genomes, have successfully captured hidden genetic diversity and facilitated the discovery of a genetic basis of important traits^16,17^.

The apple (*Malus domestica* Borkh.) is a popular temperate fruit and its domestication was driven by hybridization of different wild species and clonal propagation of genotypes with desirable traits. Among the wild species, *M. sieversii* and *M. sylvestris* are the major progenitors^18–20^. The apple genome is highly heterozygous, posing a major challenge for earlier genome assemblies^21,22^. Currently, reference-quality genome assemblies of cultivated apple are available for a double-haploid line GDDH13 (ref. ^9^), a triple-haploid HFTH1 (ref. ^23^) and a diploid cultivar ‘Gala Galaxy’^24^; for its wild relatives, only a draft genome of *M. baccata* is available^25^. In the present study, we assembled reference-grade, phased diploid genomes for the cultivated apple Gala, a top cultivar grown worldwide, and two major wild progenitors, *M. sieversii* and *M. sylvestris*. We directly sequenced the heterozygous lines and disclosed the diploid state of the genomes. We also constructed a pan-genome for each of the three *Malus* species based on 91 deeply resequenced accessions. The high-quality reference and pan-genomes enable a better understanding of the genetic basis of apple domestication, and provide valuable resources for future apple research and breeding.

## Results

### Genome assembly and homologous chromosome construction

We generated a total of 623–780× coverage of Illumina and 10x Genomics sequences and an additional 37–81× coverage of PacBio HiFi sequences for the three accessions (Supplementary Table 1). For each accession, reads were assembled into a diploid genome comprising phased scaffolds, as well as a conventional haploid consensus (Supplementary Fig. 1). The final assemblies had sizes of 1.31–1.32 Gb for diploid genomes and 652–668 Mb for haploid consensuses (Supplementary Table 2). Despite high heterozygosity rates (0.85–1.28%), all assemblies showed high contiguity, with the scaffold N50 of 3.3–4.3 Mb in diploid assemblies and 16.8–35.7 Mb in haploid consensuses (Supplementary Table 2). Using high-density genetic maps^26,27^ and genomic synteny with the published assembly^9^, 96.7–97.8% of scaffolds in haploid consensuses were successfully anchored.

**Fig. 1:**
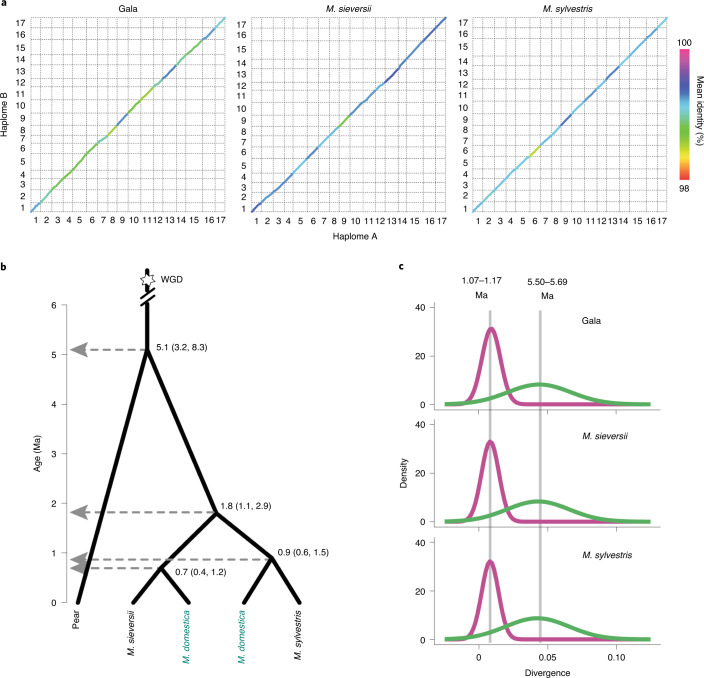
Genome and evolution of Gala, *M. sieversii* and *M. sylvestris*. **a**, Genomic synteny between homologous chromosomes (haplomes) of the diploid assemblies. Numbers indicate chromosomes. **b**, Evolutionary history and divergence time of the apple. The estimated divergence times (Ma) are inferred based on single-copy orthologous groups, and the 95% highest posterior density ranges (in the brackets) are shown at each node. The colored *M. domestica* represent the ancestral subpopulations of *M. sieversii* and *M. sylvestris* that are the direct genetic contributors of modern-day cultivated apples. WGD, whole genome duplication. **c**, Distribution of sequence divergence of intact LTR-RTs. Peak values were converted to ages based on a mutation rate of 3.9 × 10^−9^ substitutions per site per year.

**Extended Data Fig. 1:**
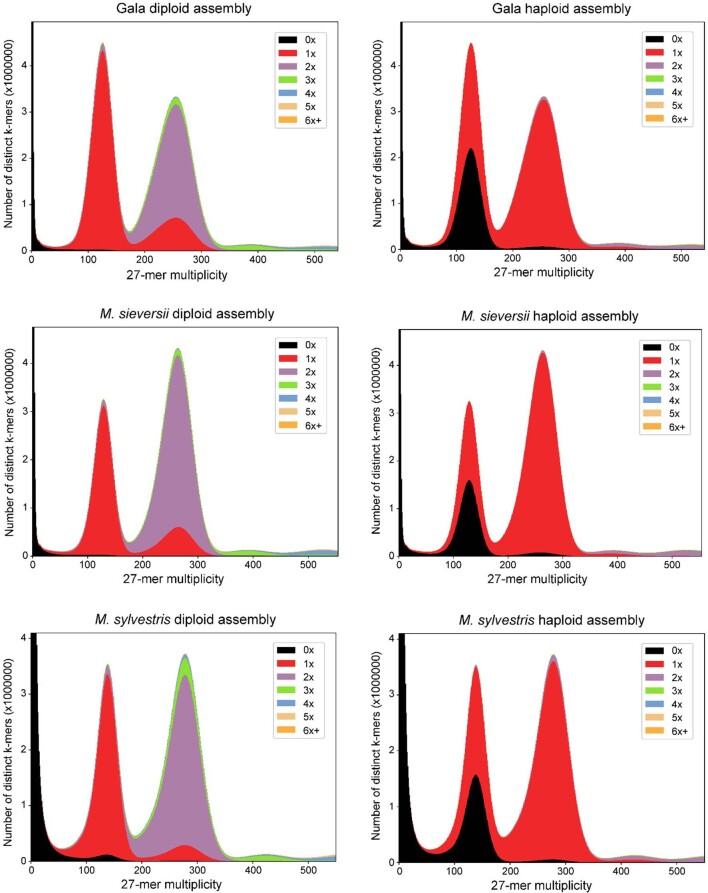
*K*-mer spectrum analysis. Comparison of 27-mer spectra between all paired-end reads (y-axis) and assemblies (x-axis) for the three apple accessions. Colors mark different copies of 27-mers in the genome assembly. The black area measures the proportion of 27-mers that were present in the sequencing reads but missing in the assemblies, which could be sequencing errors and contaminated sequences from organelles and microorganisms. The presence of a small portion of single-copy k-mer showing homozygous peak in the diploid assemblies could be due to: 1) the two alleles are nearly identical and were collapsed into one copy in the diploid assembly; and 2) the phased diploid assemblies are incomplete, with only one allele assembled for some loci.

**Extended Data Fig. 2:**
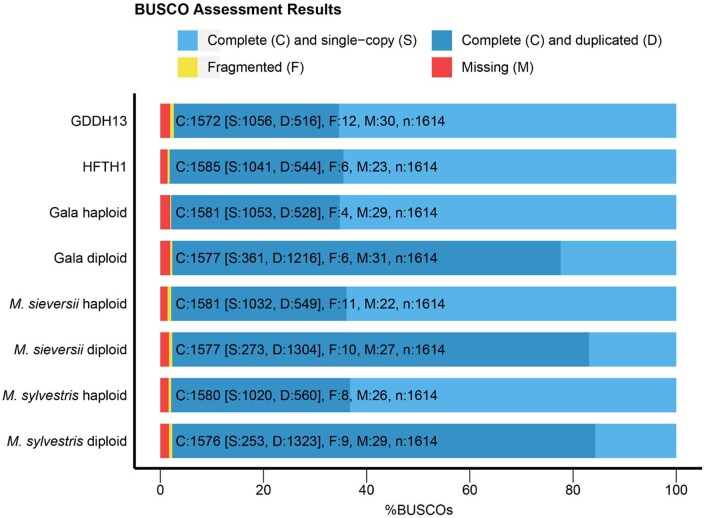
BUSCO evaluation of apple genome assemblies. Gene space coverage of apple genome assemblies was assessed using 1,614 core conserved plant genes with BUSCO (https://busco.ezlab.org/).

The diploid assemblies were approximately twice the size of the haploid consensuses, indicating that the homologous chromosomes were well captured in each assembly, which was further supported by the *k*-mer spectrum analysis (Extended Data Fig. 1). Approximately 93.7–95.5% of the phased scaffolds were separated into two nonredundant collections (a.k.a. haplomes), which were further anchored to 17 homologous chromosomes. The cumulative size of each haplome was 88.5–100.0% of the haploid consensus (Supplementary Table 2), and all three accessions showed high levels of collinearity between the two haplomes (Fig. 1a). Genome evaluation using multiple approaches confirmed the high quality of both haploid and diploid assemblies (Supplementary Note and Extended Data Fig. 2).

A total of 90,147–90,507 protein-coding genes were predicted in the diploid assemblies and 45,199–45,352 in the haploid assemblies (Supplementary Table 2). The nucleotide-binding, leucine-rich repeat genes are broadly involved in disease resistance^28^, and were found to be highly variable in the *Malus* spp. (Supplementary Note, Supplementary Table 3 and Supplementary Fig. 2).

**Extended Data Fig. 3:**
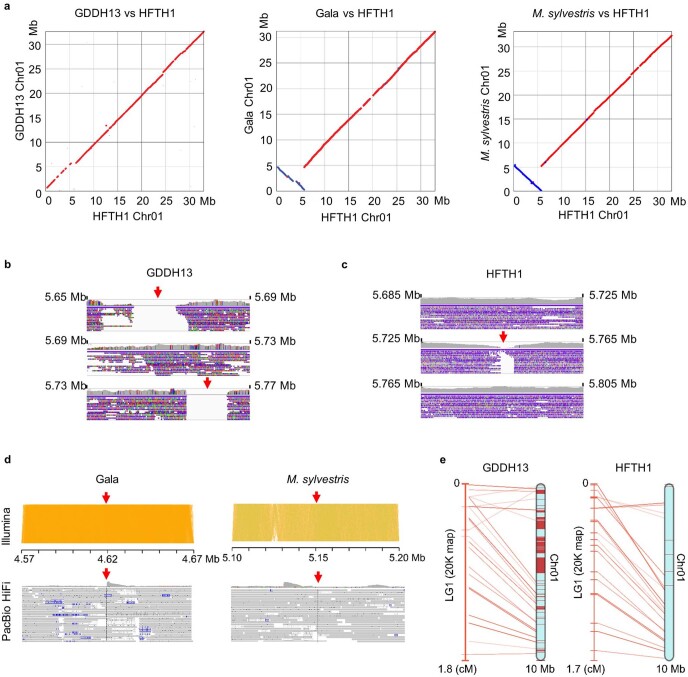
A mis-assembly identified in the genomes of GDDH13 and HFTH1. **a**, Synteny of chromosome 1 between *Malus sylvestris*, Gala, GDDH13 and HFTH1. **b** and **c**, Alignments of PacBio long reads from GDDH13 and HFTH1 to the corresponding genome assemblies indicated that the ~5-Mb inversion on chromosome 1 in GDDH13 and HFTH1 is a mis-assembly in both genomes. Breakpoints (indicated by red arrows) of the inversion in both GDDH13 and HFTH1 assemblies were surrounded by regions without PacBio long read support. **d**, Breakpoints (indicated by red arrows) of the inversion in Gala and *M. sylvestris* were well supported by both Illumina and PacBio HiFi reads. **e**, Genetic maps show inconsistency in the ~5-Mb region in both GDDH13 and HFTH1 assemblies.

Our assemblies showed an overall high collinearity with the published genomes (Supplementary Fig. 3), with an exception of a 5-Mb inversion on chromosome 1, which we found was probably a mis-assembly in both GDDH13 and HFTH1 genomes (Extended Data Fig. 3). We identified substantial variations between the haplomes, including 2,387,290, 2,591,444 and 2,929,832 single-nucleotide polymorphisms (SNPs), 363,464, 364,605 and 401,893 insertions/deletions, and 202, 343 and 330 inversions in *M. sieversii*, *M. sylvestris* and Gala, respectively (Supplementary Table 4 and Supplementary Fig. 4).

**Extended Data Fig. 4:**
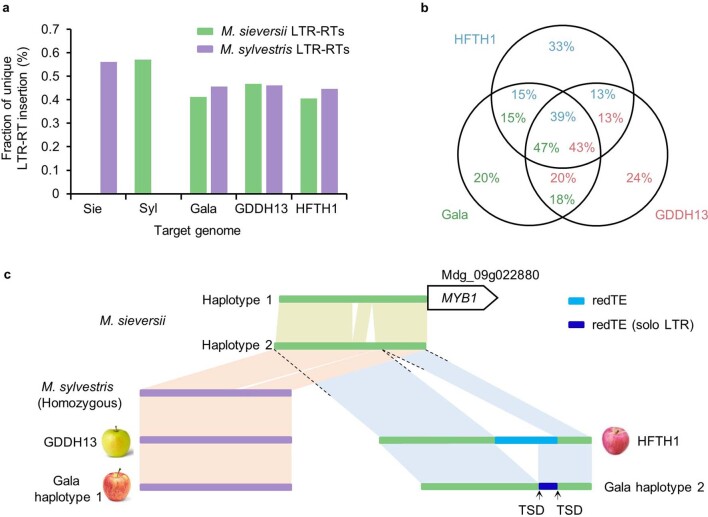
LTR-RT diversity in cultivated and wild apples. **a**, Comparison of LTR-RT insertions in different assemblies. Genomic sequences harboring LTR-RTs in *M. sieversii* or *M. sylvestris* were compared to the syntenic regions of other *Malus* assemblies to check the presence or absence of LTR-RT insertion in same regions. The percentage of unique LTR-RT insertion was calculated by dividing the number of wild species-specific LTR-RT insertions over the total number of traceable LTR-RTs in the target genome. **b**, Venn diagram showing percentages of positionally conserved LTR-RT insertions in the three cultivated apple genomes. **c**, Inheritance of the *MYB1* (Mdg_09g022880) locus in the cultivated apples and its contribution to apple fruit skin color. TSD, target site duplication.

Approximately 58.7–59.4% of the three apple genomes were repeat sequences, similar to those in genomes of GDDH13 and HFTH1 (Supplementary Table 5 and Supplementary Fig. 5). We found that two long terminal repeat retrotransposon (LTR-RT) bursts occurred during apple evolution, with the older one taking place before speciation of apple and pear^29^ and the recent one happening before the time when *M. sylvestris* and *M. sieversii* diversified into subpopulations, respectively (Fig. 1b,c and Supplementary Figs. 6 and 7). Evolution of LTR-RTs after repeated bursts may have created abundant genetic diversity among species. A notable example is the redTE retrotransposon, which translocated into the *MYB1* locus in some apple cultivars that caused red fruit skin color^23^. We found that redTE was present only in *M. sieversii* and *M. domestica*, and its presence as a short and heterozygous form in Gala probably caused *MYB1* allele-specific expression and consequently the yellowish-red fruit skin color of Gala (Supplementary Note and Extended Data Fig. 4).

### Genomic origin of the cultivated apple

**Fig. 2:**
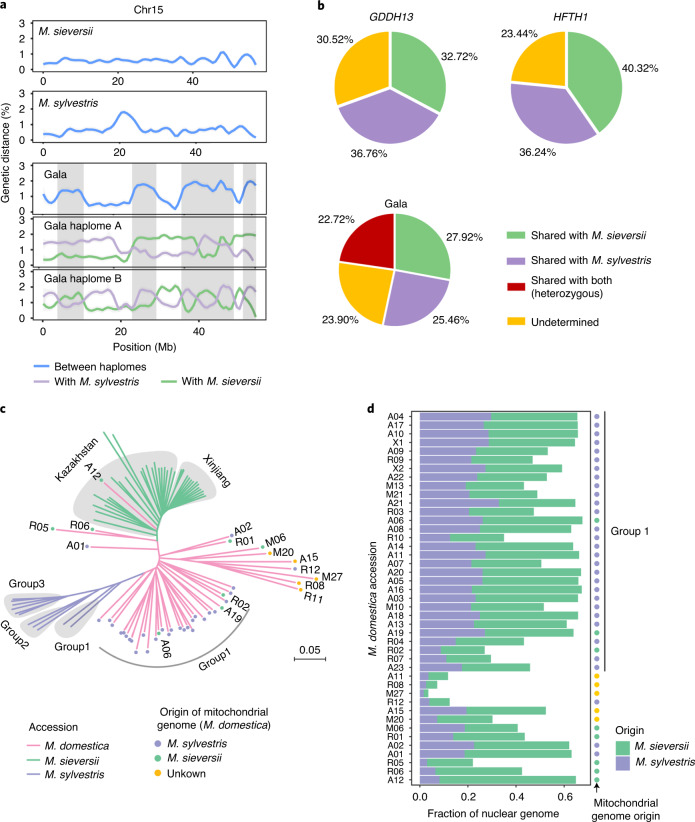
Genetic contribution of wild progenitors to the cultivated apple. **a**, Genetic distance between the two haplomes of the diploid assemblies (blue lines in the upper three panels), and between Gala haplomes and the consensus assemblies of either *M. sieversii* (green lines in the lower two panels) or *M. sylvestris* (purple lines). The highlighted areas indicate genomic regions with elevated divergence between Gala haplomes. Chromosome 15 (Chr15) is shown here whereas other chromosomes are shown in Extended Data Figs. 6 and 7. **b**, Summary of genomic contributions of the two wild progenitors to Gala, GDDH13 and HFTH1. **c**, Neighbor-joining phylogeny of 91 apple accessions based on 9,988,777 biallelic SNPs of the nuclear genome. Colored dots on cultivated apple branches indicate the origin of mitochondrial genomes. **d**, Proportion of genomes of the cultivated apple that originated from the two wild progenitors. Colored dots indicating the origin of mitochondrial genomes are the same as in **c**.

**Extended Data Fig. 5:**
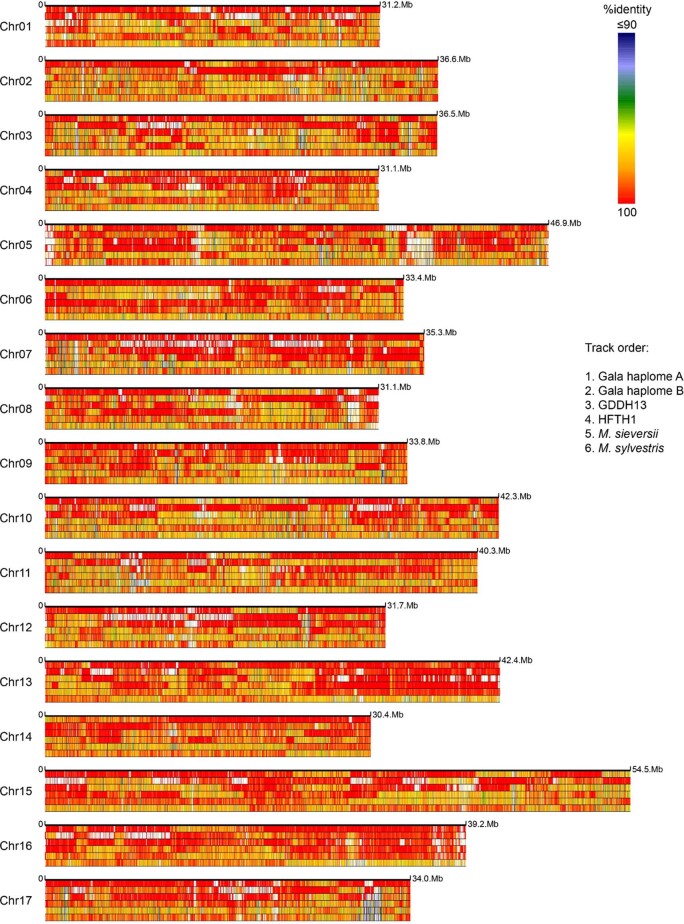
Multiple alignments of six genome assemblies using the Gala consensus genome as the reference. Scale bar represents the percent identity of the aligned regions between each assembled genome and the Gala consensus genome.

**Extended Data Fig. 6:**
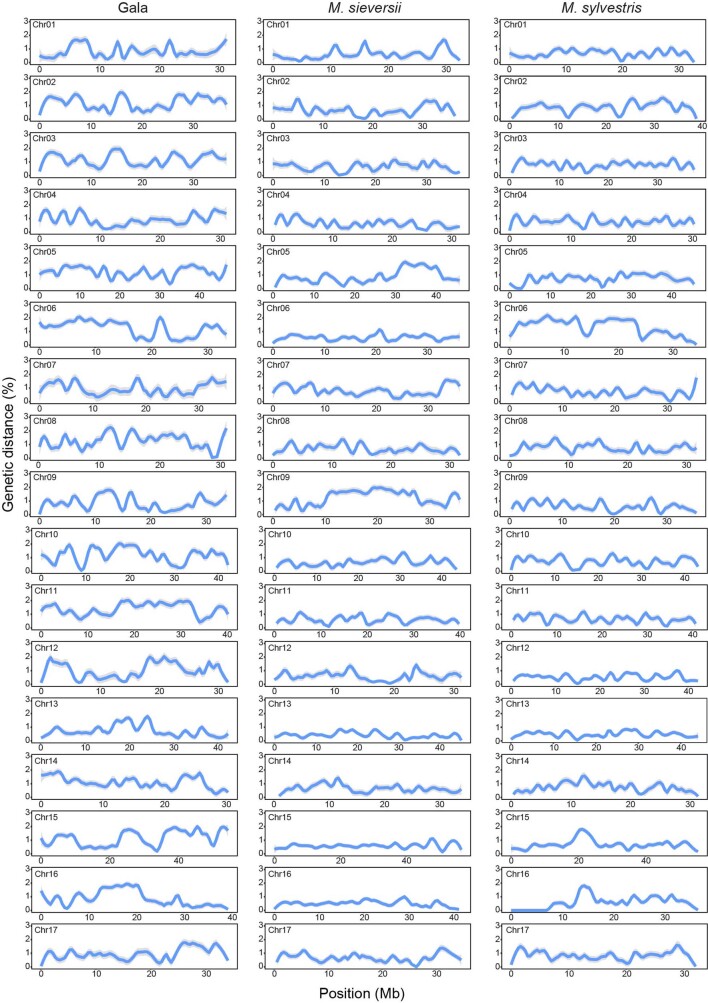
Genetic distance between the two haplomes of the diploid assemblies. Based on the alignments of syntenic regions between the two haplomes, genetic distances were calculated using distmat (http://www.bioinformatics.nl/cgi-bin/emboss/distmat) with the Jukes-Cantor correction.

**Extended Data Fig. 7:**
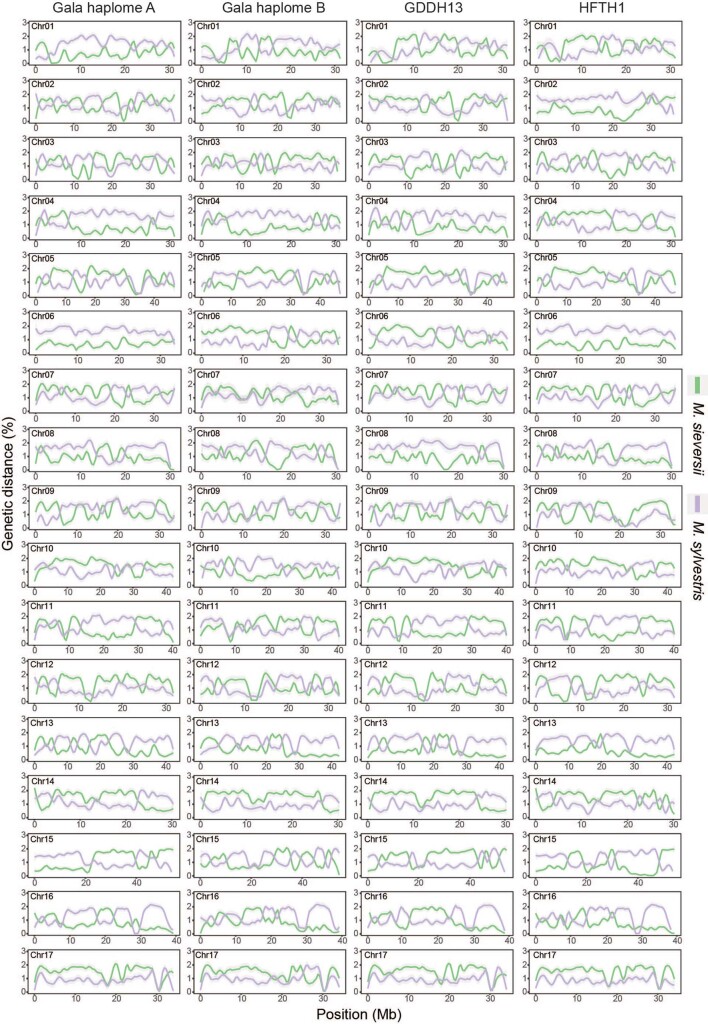
Genetic distance between the Gala haplomes and the consensus assemblies of *M. sieversii* (green) and *M. sylvestris* (purple). Based on the alignments of syntenic regions between each genome/haplome and genomes of the two wild progenitors, *Malus sieversii* and *M. sylvestris*, genetic distances were calculated using distmat (http://www.bioinformatics.nl/cgi-bin/emboss/distmat) with the Jukes-Cantor correction.

Syntenic regions of *M*. *sieversii* and *M*. *sylvestris*, with respect to the Gala genome, were genetically divergent and formed mutually distinguishable blocks where the boundaries of the discrete blocks indicated the possible recombination sites in Gala (Extended Data Fig. 5). The genetic distance between the two haplomes in Gala fluctuated across chromosomes and the average divergence was higher than that in the two wild species (Extended Data Fig. 6). We found that the highly divergent regions between the two Gala haplomes corresponded to a hybrid origin of the two alleles, whereas less divergent regions underlined homozygous alleles that originated from either *M. sieversii* or *M. sylvestris* (Fig. 2a). We inferred that the portion of cultivated apple genomes probably derived from *M. sieversii* ranged from 28% to 40%, and from *M. sylvestris* 25–37% (Fig. 2b). The heterozygous Gala genome encoded 23% of the sequence with hybrid ancestry, indicating that a considerable portion of the Gala genome has preserved genetic information from both progenitors (Fig. 2b). Moreover, 28% of syntenic regions among the three cultivated apples were derived from different progenitors (Extended Data Fig. 7), suggesting frequent recombination in apple genomes.

To make a population-scale inference, we collected 91 accessions (43 *M. domestica*, 37 *M. sieversii* and 11 *M. sylvestris*), including 71 from our previous study^20^. Of these 91 accessions, 59 were further or newly deep sequenced, yielding a total of 2.7 Tb data with an average depth of 59.3× (Supplementary Table 6). A total of 9,988,777 biallelic SNPs and 323,047 structure variants (SVs) were identified. Phylogenetic and principal component analyses indicated that *M. sieversii* and *M. sylvestris* accessions clustered into clearly separated monophyletic clades, whereas *M. domestica* accessions clustered within paraphyletic groups (Fig. 2c and Supplementary Fig. 8). Estimation of genomic origin in these accessions revealed substantial genetic contribution of the two wild progenitors to the cultivated apple (Fig. 2d and Supplementary Table 7). Phylogeny based on the assembled mitochondrial or chloroplast genomes showed that 30 of the 43 *M. domestica* accessions clustered with *M. sylvestris* (Supplementary Fig. 9), indicating substantial maternal pedigree from *M. sylvestris* in the cultivated apple.

### Selective sweeps underlying trait inheritance from different progenitors

**Fig. 3:**
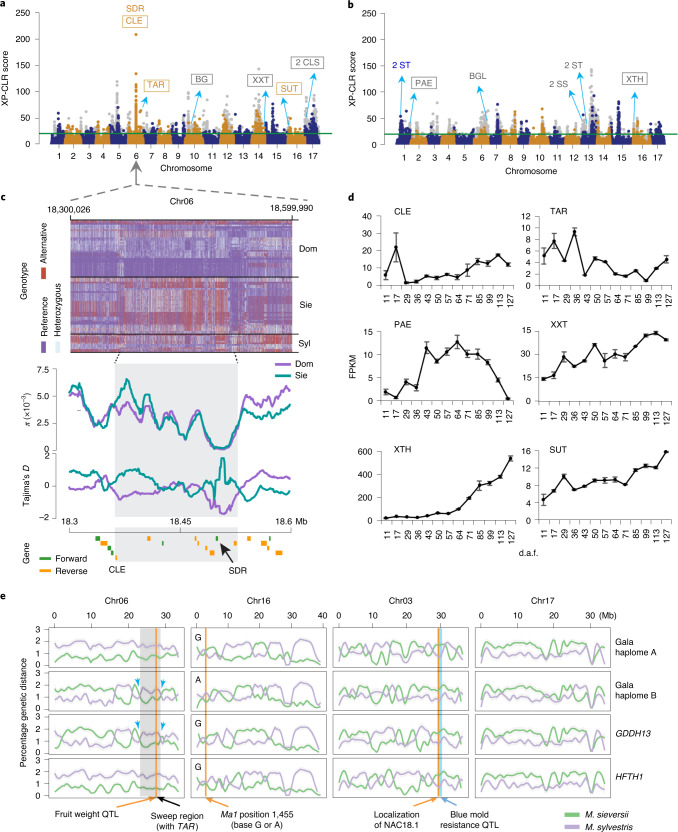
Selective sweeps in the cultivated apple genome. **a**,**b**, Selective sweeps and candidate genes in cultivated apple compared with *M. sieversii* (**a**) and *M. sylvestris* (**b**). Chromosomes are painted with either dark-blue or orange, whereas the gray points indicate genomic regions that originated from the other wild progenitor rather than the compared one. Green lines define the top 1% XP-CLR scores. Genes inside boxes were differentially expressed during apple fruit development. **c**, SNP genotype, nucleotide diversity (*π*) and Tajima’s *D* in the specified genome region of the three *Malus* populations. **d**, Expression profiles of selected genes during apple fruit development. The mean of FPKM (fragments per kilobase of exon per million mapped fragments) and its s.e. derived from three biological replicates are shown. **e**, Genetic distance showing the origin of the selected chromosomes from the two wild progenitors. Shade in chromosome 6 indicates the region originated from *M. sieversii*, which is surrounded by recombination spots indicated by blue arrows.

The cultivated apple bears many traits that are selected during domestication. Some traits, including fruit weight, firmness, sweetness and tartness, resemble either of the two progenitors^20^. We identified a total of 1,633 and 1,504 genomic regions under selection for the comparisons of *M. domestica* with *M. sieversii* and *M. sylvestris*, respectively, with cumulative sizes of 18.5 and 18.9 Mb, and harboring 1,400 and 1,259 genes (Fig. 3a,b and Supplementary Tables 8 and 9). Among the 3,137 selective regions, 1,295 and 1,121 shared genome ancestry with *M. sieversii* and *M. sylvestris*, respectively. The genomic region with the highest selection score (XP-CLR^30^) contained nine genes on chromosome 6 (Fig. 3c), including one CLAVATA3/ESR (CLE)-related gene, which was upregulated during apple fruit development (Fig. 3d), and one encoding a short-chain dehydrogenase/reductase (SDR). The CLE family comprises a major group of signaling peptides and displays diverse functions in plants^31^. A notable example is *CLV3*, the loss-of-function alleles of which can increase tomato yield by regulating fruit size^32^. SDR is involved in the production of alcohol-related substrates, which are important compounds contributing to apple fruit aroma^33^.

The cultivated apple inherited its fruit size mainly from *M. sieversii*^18^. A fruit weight quantitative trait locus (QTL)^34^ on chromosome 6 (24–29 Mb) was found in a region surrounded by recombination spots, whereas only the *M. sieversii* allele was inherited by the cultivated apple (Fig. 3e), suggesting a strong selection on this region during domestication. A selective sweep containing a tryptophan aminotransferase-related (*TAR*) gene was found within this QTL (Fig. 3a,e). *TAR* is involved in indole-3-acetic acid biosynthesis and its expression was high at early fruit developmental stages (Fig. 3d). It is of interest that overexpression of the *TAR* homolog in wheat enhanced grain yield^35^, indicating a potential role of *TAR* in apple fruit size regulation.

Wild and cultivated apples show a remarkable difference in fruit acidity. The *Ma1* gene encoding a malate transporter was identified as a major locus controlling apple fruit acidity^36^. A mutation (G to A) at base 1,455 of the *Ma1* coding sequence leads to protein truncation that is largely responsible for the low fruit acidity in apples^37^. We found that the frequency of the ‘A’ allele was low in *M. sylvestris* (4.5%), but dramatically increased in *M. sieversii* (33.3%) and the cultivated apple (55.6%), consistent with fruit acidity levels of the cultivated apple being comparable to *M. sieversii* but substantially lower than *M. sylvestris*^20^. These data suggest that the low acidity ‘A’ allele of the cultivated apple is inherited mainly from *M. sieversii*, as is the case in Gala (Fig. 3e). Moreover, a much higher proportion of the cultivated apple (66.7%) harbored both alleles than *M. sieversii* (44.4%) and *M. sylvestris* (9.1%), consistent with a heterozygous locus in cultivars being responsible for the moderate acidity favored by consumers^36^.

The firm texture of cultivated apple fruits is mainly contributed by *M. sylvestris*^18^. The apple *PG1* (polygalacturonase 1) gene harbors two different alleles with one associated with crispy texture and the other with mealy texture^38^. Most *M. sieversii* accessions harbored the homozygous mealy texture-associated allele, whereas *M. sylvestris* accessions mainly comprised the homozygous crispy texture-associated allele (Supplementary Fig. 10). Cultivated apples harbored a much higher percentage of heterozygous genotype of *PG1*, suggesting a favorable fruit firmness achieved through hybridization of wild progenitors during domestication. In addition, a nonsynonymous SNP (C/A) in the transcription factor gene *NAC18.1* was found to be associated with fruit firmness^39^. Overexpression of the *NAC18.1* haplotype containing the ‘C’ allele resulted in firmer fruit than that with the ‘A’ allele^39^. We found that the ‘C’ allele was fixed in *M. sylvestris* (100%) and predominant (82.0%) in the cultivated apple, whereas in *M. sieversii* the ‘A’ allele was predominant (67.0%), consistent with the genomic region harboring *NAC18.1* being derived from *M. sylvestris* in all three cultivars (Fig. 3e). Moreover, the major blue mold resistance QTL^40^, which was also derived from *M. sylvestris*, co-localized with this fruit firmness locus (Fig. 3e). Many *M. sieversii* accessions are resistant to blue mold, whereas most cultivars are susceptible, indicating a possible hitchhiking effect caused by fruit firmness selection in the cultivated apple during domestication.

Gala, GDDH13 and HFTH1 all inherited chromosome 17 largely from *M. sylvestris* (Fig. 3e). This chromosome harbored selective sweeps overlapping with QTLs associated with apple polyphenol content^41,42^ and containing genes related to cell-wall biosynthesis, including two encoding cellulose synthases (CLSs). One CLS gene and several other cell-wall-related genes in sweep regions of other chromosomes (for example, pectin acetylesterase (PAE), xyloglucan 6-xylosyltransferase (XXT) and xyloglucan endotransglucosylase/hydrolase (XTH)) were all upregulated during fruit development (Fig. 3a,d), therefore probably contributing to apple fruit firmness. In addition, we identified sweeps that contained genes associated with fruit sweetness, including those encoding sugar/sucrose transporter (ST/SUT), sucrose synthase (SS) and β-glucosidase/galactosidase (BG/BGL) (Fig. 3a,b,d).

### Population structure and demographic history of apples

**Fig. 4:**
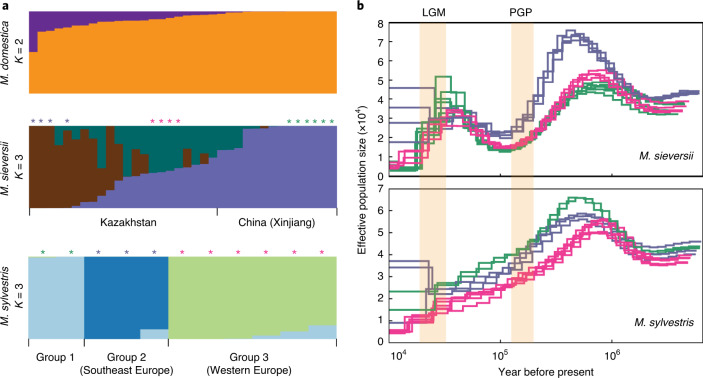
Population structure and demographic history of the apple. **a**, Population structure of the cultivated apple and the two wild progenitors, *M. sieversii* and *M. sylvestris*. Only clusters (*K*) with the highest *ΔK* are shown. **b**, Effective population size of *M. sieversii* and *M. sylvestris*. Colored lines correspond to accessions labeled with the same colors as the asterisks in **a**. Highlighted areas represent the time interval of the PGP and the LGM.

Population structure analysis indicated two obvious gene pools in apple cultivars (Fig. 4a). The *M. sieversii* accessions were collected from Kazakhstan and Xinjiang, China, two sides of the Tian Shan mountains. Under the optimal number of clusters (*K* = 3; Supplementary Fig. 11), the Xinjiang accessions showed homogeneous genetic background with low levels of introgression from other populations. In contrast, the Kazakhstan accessions consisted of two gene pools and some accessions were under intensive introgression (Fig. 4a). Accessions with high levels of introgression were sampled in places closer to the route of the ancient Silk Road (Supplementary Fig. 12), suggesting that massive introgression may have occurred during westward dissemination of the apple along the Silk Road^18^. *M. sylvestris* comprised three geological subpopulations (Fig. 4a), two of which are known to be from south-east Europe and western Europe. Introgressions between these subpopulations were rare, confirming geographic isolation among subpopulations.

Inference on demographic history indicated a decline of effective population size (*N*_e_) starting ~0.9 million years ago (Ma) for both *M. sieversii* and *M. sylvestris* (Fig. 4b). The contraction of *N*_e_ occurred during the ice age known as quaternary glaciation (2.58 Ma to the present), and was common in many other plants^43–45^. The *N*_e_ of *M. sieversii* reached the bottom and then started to rebound 128 to 123 thousand years ago (ka), which corresponded to the termination of the penultimate glacial period (PGP) (130 to 113 ka) and the onset of the last interglacial period (130 to 115 ka), during which deglaciation took place^46^ (Fig. 4b). The second bottleneck for *M. sieversii* started 40 to 30 ka, right before the onset of the last glacial maximum (LGM)^47^ (33 to 19 ka) which caused an inhospitable environment for living organisms. The *M. sylvestris* population continued to decline in the long and distant past, indicating that climate fluctuation may have led to continuous population contraction of *M. sylvestris*. This coincides with the hypothesis that quaternary glaciation has narrowed down and fragmented the geological range of *M. sylvestris*, therefore leading to the emergence of geographic subpopulations^48^.

### Pan-genomes of cultivated and wild apples

**Fig. 5:**
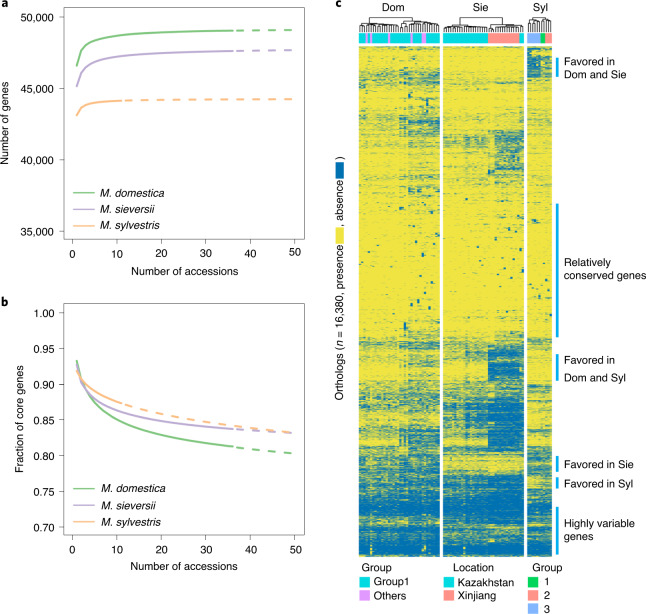
Pan-genomes of the cultivated apple and the two wild progenitor species. **a**,**b**, Modeling of pan-genomes (**a**) and core genomes (**b**) of the three *Malus* species. The size of pan-genome or core genome at each specified number of accessions was modeled with power–law distribution. The solid line shows fitting to the maximum number of sampled accessions, whereas the dashed line indicates the exploration of the fitting. **c**, PAV of pan-genome orthologues in *M. domestica*, *M. sieversii* and *M. sylvestris*. Only orthologues containing genes absent in at least one accession were shown. Dom, *M. domestica*; Sie, *M. sieversii*; Syl, *M. sylvestris*.

**Extended Data Fig. 8:**
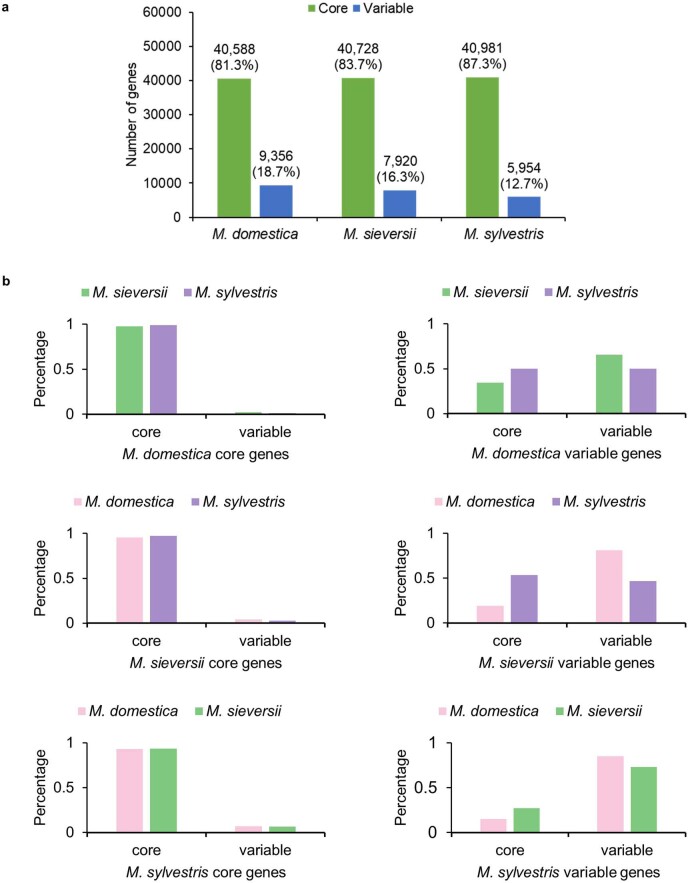
Core and dispensable genes of *Malus domestica*, *M. sieversii* and *M. sylvestris*. **a**, Core and variable genes in each species; **b**, Conservation of core and variable genes across different species. Each bar graph shows the percentage of overlapped core and variable genes in different species.

We constructed pan-genomes for the three *Malus* species separately by de novo assembly of the resequencing data for each accession (Supplementary Fig. 13). Consequently, a total of 89-, 212- and 141-Mb nonredundant, nonreference sequences harboring 1,736, 3,438 and 2,104 new genes were identified for *M. sylvestris*, *M. sieversii* and *M. domestica*, respectively, which brought pan-genomes containing 46,935, 48,648 and 49,944 protein-coding genes. Modeling of pan-genome sizes suggested closed/saturated pan-genomes for all three species (Fig. 5a). Gene Ontology term enrichment analysis on the new genes suggested that genes associated with pollination, signal transduction and response to stress were highly enriched (Supplementary Fig. 14). *M. domestica* had the largest pan-genome due to genetic introgression from wild species during domestication, whereas *M. sylvestris* had the smallest, congruent with the constant bottleneck in its demographic history (Fig. 4b). The fraction of core genes in the three pan-genomes (81.3–87.3%; Extended Data Fig. 8a) was higher than that in the annual plants^13,49–55^ (35–81%). The core genomes of the three species showed a different trend, with *M. domestica* having the lowest fraction of core genes (Fig. 5b). Core genes were relatively conserved across species, whereas variable genes showed high plasticity in different species (Extended Data Fig. 8b).

The presence/absence variation (PAV) pattern of pan-genomes can serve as an indicator of genes under selection^13^. To identify cross-species PAVs, we clustered the three pan-genomes into 69,411 orthologues (Supplementary Tables 10 and 11). The phylogeny of the apple accessions inferred using PAVs (Supplementary Fig. 15) was consistent with the SNP phylogeny (Fig. 2c). We identified 851 orthologues that were favored in both *M. domestica* and *M. sylvestris* compared with *M. sieversii*, 316 favored in both *M. domestica* and *M. sieversii* compared with *M. sylvestris*, and 17 exclusively favored in *M. domestica* (Fig. 5c and Supplementary Table 12). The much fewer uniquely favored genes in *M. domestica* than those shared with one of the wild progenitors suggests that many trait-associated genes in the cultivated apple may have already been functionally divergent in the wild progenitors, whereas domestication may have created relatively few innovations on the selection of genes. PAVs of disease resistance genes as well as self-incompatibility genes were found in different groups of favored genes, congruent with their rapid evolution. Several cell-wall biogenesis-associated genes were favored in both *M. sylvestris* and *M. domestica* compared with *M. sieversii*, whereas genes encoding a chitinase and three WRKY transcription factors, with *Arabidopsis* homologs that conferred drought tolerance, were favored in both *M. sieversii* and *M. domestica* when compared with *M. sylvestris* (Supplementary Table 12).

### Widespread ASE during apple fruit development

**Fig. 6:**
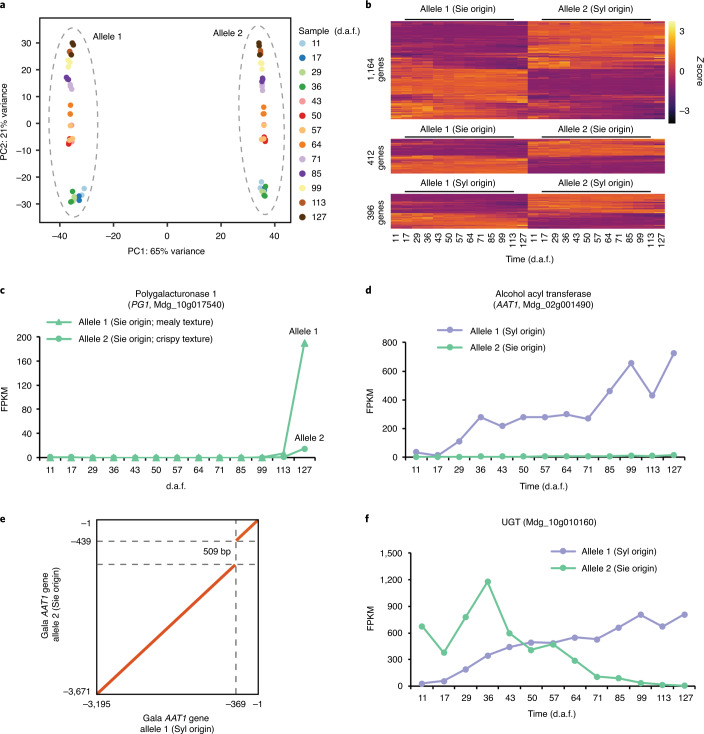
Allele-specific gene expression in Gala fruit. **a**, Principal component (PC) analysis of Gala fruit allele expression profiles at 13 different developmental stages. **b**, Allele expression pattern of genes with defined allele ancestry and showing ASE in at least five fruit stages. Sie, *M. sieversii*; Syl, *M. sylvestris*. **c**, Expression of the *PG1* two alleles. **d**,**e**, Expression (**d**) and structure variation (**e**) of the two alleles of *AAT1* in Gala. **f**, Expression of the two alleles of the gene encoding a UGT.

**Extended Data Fig. 9:**
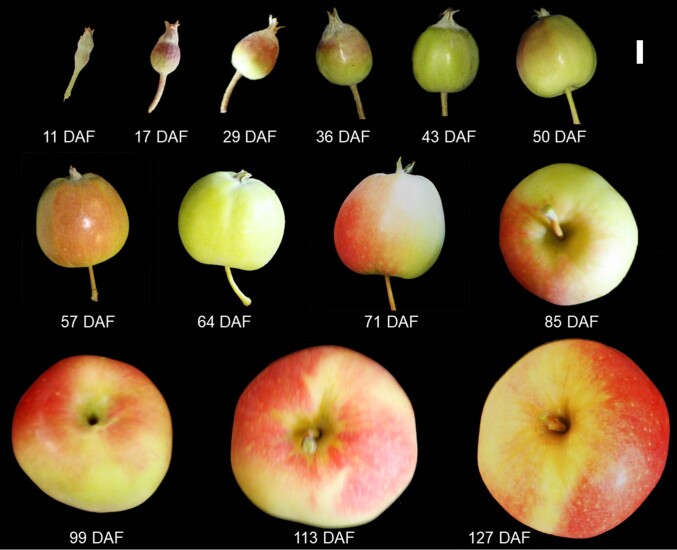
Fruits of Gala at 13 different development stages. DAF, days after full bloom. Scale bar represents 1 cm.

**Extended Data Fig. 10:**
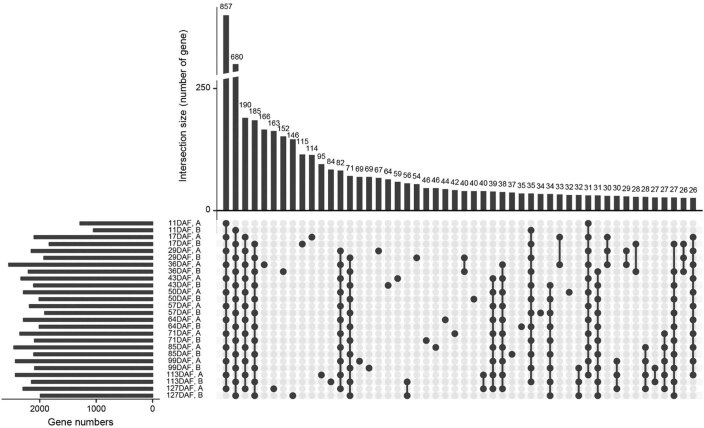
Allele-specific gene expression during Gala fruit development. UpSet plot (https://www.ars-grin.gov/), showing overlap of ASE genes in different stages. Only top 50 categories are shown. A, the dominant allele was from haplome A. B, the dominant allele was from haplome B. DAF, days after full bloom.

The phased diploid genomes allowed investigation of allele-specific expression (ASE) at a high resolution. We performed transcriptome profiling of Gala fruits at 13 different stages throughout fruit development (Supplementary Table 13 and Extended Data Fig. 9). Principal component analysis showed that the fruit transcriptomes were mainly shaped by genome-wide ASE, and secondarily by expression of genes at different developmental stages (Fig. 6a). We found that 8,569 (19% of total) genes showed ASE in Gala fruits, with 79% of them having allele imbalance at multiple stages (Supplementary Table 14). Most ASE genes had a dominant allele with expression that was consistently higher than that of the other allele at all stages showing ASE, whereas switch of allele dominance was observed for only 112 genes (Fig. 6b and Extended Data Fig. 10). Among the ASE genes, those with two alleles derived from different progenitors were overrepresented (Fisher’s exact test; *P* = 9.4 × 10^−49^); however, expression of different types of ASE genes classified based on their allele ancestry did not show bias toward either progenitor (Fig. 6b), reinforcing the critical contribution of both progenitors in gene expression regulation in cultivated apples. We found that SVs between the two Gala haplomes located upstream of genes were significantly closer to ASE genes than to other genes (Mann–Whitney *U*-test; *P* = 4.5 × 10^− 23^; Supplementary Fig. 16a), suggesting that SVs could function as *cis* variations that drive ASE in Gala.

Many of the ASE genes were associated with fruit development and quality, including those encoding ACC oxidases and RIN-like MADS-box transcription factor for fruit ripening, OVATE family proteins for fruit shape, cell-wall metabolic genes involved in fruit texture, and genes associated with biosynthesis of phytohormones, flavonoids and aroma volatiles (Supplementary Fig. 16b). The Gala genome harbored both *PG1* alleles, the expression of which was low and indistinguishable at most of the fruit developmental stages. However, at the ripening stage (127 d after full-bloom (d.a.f.)), the mealy texture-associated allele was expressed to a much higher level (Fig. 6c), consistent with the relatively soft fruit texture of Gala. The aroma of ripe apples arises from a complex mixture of volatile compounds including esters^33^. The apple *AAT1* encoding an alcohol acyl transferase is the major gene controlling ester production^56^. The *AAT1* gene in Gala displayed strong ASE, with the *M. sieversii*-derived allele expressed at much lower levels than the *M. sylvestris*-originated allele at most developmental stages (Fig. 6d and Supplementary Fig. 16c). We identified an insertion of a 509-bp miniature inverted-repeat transposable element–like sequence in the upstream region of the *M. sieversii*-derived allele (Fig. 6e). Such insertion was also found in the cultivar ‘Granny Smith’ that harbors homozygous alleles and produces low levels of ester, demonstrating its association with *AAT1* expression and ester production, and suggesting that Gala ester production is mainly attributed to the *M. sylvestris*-originated allele. Flavor-related volatiles in developing fruits can be modified by UDP-glycosyltransferases (UGTs), which are also involved in the biosynthesis of anthocyanin and flavonols^57^. Among the 69 UGTs showing ASE in Gala fruits, 30 were dominated by the allele originating from *M. sieversii*, whereas 18 were dominated by the *M. sylvestris*-derived allele. Unlike other UGTs with one allele dominant throughout fruit development, the two alleles of the UGT91 subfamily gene *Mdg_10g010160* showed distinct dominant patterns. The *M. sieversii*-originated allele dominated at the early developmental stages whereas the *M. sylvestris*-derived allele dominated during ripening (Fig. 6f). As the transcriptional regulation of fruit development and ripening is tightly controlled^58^, such transition of allele dominance for *Mdg_10g010160*, as well as 111 other genes with a similar dominance pattern, may indicate an allele-level interplay that regulates apple fruit development and ripening.

## Discussion

Unlike many annual crops, apple domestication was mainly driven by hybridization of different wild species. Traits introgressed in the hybrid are often not fixed and could be lost when propagated by seeds. Understanding of the molecular basis of trait variability, which requires the knowledge of the diploid alleles, is critical for fixation of desirable traits in apple breeding. We generated reference quality and haplotype-resolved diploid genomes of Gala, a top apple cultivar in the world, frequently used as a parental line in apple breeding^40^, and the two major wild progenitors, *M. sieversii* and *M. sylvestris*. The two wild progenitors showed distinct demographic histories, with *M. sieversii* recovering its population size after the first bottleneck whereas populations of *M. sylvestris* continued to decline, explaining why *M. sylvestris* has been considered as an endangered species in Europe^59^. At least 28% of syntenic genome regions in Gala, GDDH13 and HFTF1, and 23% of the Gala genome harbor alleles derived from different progenitors. Such high level of heterozygosity may have a twofold impact on the genome: (1) masking the deleterious effect of recessive alleles, which can create heterosis^10^; and (2) causing ASE. We found at least 19% of Gala genes showed ASE during its fruit development, and these genes were involved in diverse biological processes related to fruit quality. It is worth noting that the role of ASE in apple domestication has long been ignored, probably limited by the lack of diploid phased and wild progenitor genome sequences. Hence, the high-resolution fruit ASE analysis in the present study filled an important gap in our understanding of fruit trait regulation and domestication of cultivated apples.

Fruit weight, acidity, sweetness, firmness and polyphenol content are well-known domestication traits that remain as the main targets of current apple breeding programs. Our results unraveled many previously unreported trait-associated genes that are located at selective sweeps. Analysis of the origin of these genes, as well as others with established roles, provided new insight into the evolution of trait-associated alleles during apple domestication. Moreover, we found that numerous trait-associated alleles have been fixed or nearly fixed in the cultivated apple and one of the wild progenitors. Such a pattern is different from that in many other crops, for which major domestication trait-associated alleles are fixed only in the cultivars. This demonstrates the uniqueness of apple domestication that was mainly driven by hybridization of different wild species, rather than by evolution to form an independent lineage or species.

Pan-genomes complement reference genomes by including nonreference genes present in a population. A total of 1,736–3,438 new genes were discovered in the 3 *Malus* populations. The observation of 12.7–18.7% of genes in the pan-genomes showing PAVs highlights the genetic plasticity in apple populations. Unlike annual crops such as the tomato^13^, the pan-genome size of the cultivated apple is larger than that of wild progenitors, possibly due to the outcrossing nature and extensive introgression from wild species. This distinctive feature suggests that introgression of new genes/alleles is possibly a hallmark of crops domesticated through hybridization. PAV analysis identified hundreds of genes that were uniquely favored in the two wild progenitors, which were further preserved in the cultivated population. These genes, along with the identified new genes, provide useful resources for understanding the genetic basis of various apple traits and for facilitating future apple breeding.

## Methods

### Library construction and sequencing

For de novo assemblies, young leaves from plants of *M. domestica* cv. Gala (PI 392303), *M. sieversii* (PI 613981) and *M. sylvestris* (PI 633825), grown at the apple germplasm repository of the Plant Genetic Resources Unit, USDA-ARS, were collected and used for DNA extraction with the DNeasy Plant Mini Kit (QIAGEN). PCR-free paired-end and mate-pair libraries were constructed using the Illumina Genomic DNA Sample Preparation kit and the Nextera Mate Pair Sample Preparation kit, respectively, following the manufacturer’s instructions (Illumina). All libraries were sequenced on an Illumina HiSeq 4000 system with the paired-end mode. In addition, high-molecular-weight DNA was prepared and used to construct linked read libraries using the 10x Genomics Chromium System and PacBio SMRTbell libraries using SMRTbell Express Template Prep Kit 2.0, following the manufacturers’ protocols. The 10x Genomics libraries were sequenced on an Illumina HiSeq X system with the paired-end mode. SMRTbell libraries were sequenced on a PacBio Sequel II system and consensus reads (HiFi reads) were generated using ccs software (https://github.com/pacificbiosciences/unanimity) with the parameter ‘-minPasses 3’. For genome resequencing, sample preparation and library construction and sequencing were conducted as previously described^20^.

To assist gene prediction, RNA-sequencing (RNA-seq) data were generated from samples of fruit flesh, peel, flowers and leaves for each accession, and also from fruit samples of Gala collected at 11, 17, 29, 36, 43, 50, 57, 64, 71, 85, 99, 113 and 127 d.a.f., respectively, with three biological replicates except for 11 d.a.f., which had two. Total RNA was extracted using the RNeasy mini kit (QIAGEN). Strand-specific RNA-seq libraries were prepared using the protocol described in Zhong et al.^60^ and sequenced on a HiSeq 4000 system using the paired-end mode.

### Genome assembly

Genomes of the three accessions were first assembled with DeNovoMAGIC3 (NRGene) using Illumina paired-end and mate-pair, and 10x Genomics reads. The algorithm of the software was elaborated elsewhere^61^. For each accession, a phased diploid assembly, which comprised scaffolds each of which originated from a single (phased) allele, and an unphased consensus assembly, which comprised scaffolds containing collapsed sequences from the two alleles of each genome locus, were generated.

A pipeline was developed to validate and improve the assemblies based on the mapping of mate-pair reads and collinearity with the high-quality GDDH13 genome^9^ (Supplementary Fig. 1). For each accession, first, the mate-pair reads were aligned to the assembly using BWA^62^ (v.0.7.16a-r1181) to derive physical coverage of the assembly. Gaps at which the physical coverage was less than half of that in the 2-kb flanking regions or <20 were recorded. Second, we evaluated the reliability of connecting the two scaffolds at each recorded gap through: (1) aligning the 2-kb flanking sequences to the GDDH13 genome—the connection was considered valid if the two sequences located within a 100-kb window on the GDDH13 genome; and (2) anchoring the two scaffolds to high-density genetic maps^26,27^. The connected scaffolds consistent with the genetic maps were considered valid. Scaffolds with invalid connections were broken. Last, the resulting final scaffolds were re-anchored using ALLMAPS^63^ with the genetic maps and syntenic information with the GDDH13 genome. Additional steps were taken to validate scaffolding of phased diploid assemblies, because the existence of both alleles would challenge the assignment of mate-pair reads. Therefore, we first performed all-versus-all BLAST (identity >95%) of each of the three diploid assemblies. Scaffolds contained by or overlapped (>50%) with others were separated and formed a collection of alternative alleles. Each collection (comprising primary or alternative alleles) was validated separately and re-anchored into pseudomolecules using the procedure described above.

To further validate and improve the assemblies, we generated 37–81× PacBio HiFi reads for the three accessions. These long (~12 kb) and highly accurate (>99%) HiFi reads were assembled using Hifiasm (https://github.com/chhylp123/hifiasm) and HiCanu^64^, respectively. Two independent pipelines were developed to improve the quality of DeNovoMAGIC haploid and diploid genomes with the assemblies from HiFi reads (Supplementary Fig. 1). For each haploid genome, we first aligned the Hifiasm-assembled contigs to the DeNovoMAGIC haploid genome using MUMMER4 package^65^, and the reciprocal best alignments were identified using a delta-filter program (with the parameter ‘-g, -l 10000’) in the MUMMER4 package. Based on the alignments, gaps in the DeNovoMAGIC haploid assembly were filled with sequences from the Hifiasm assembly. The gap-filled haploid assembly was further processed to remove redundant scaffolds (coverage >0.9 and identity >95%). The final haploid assembly was then used for the second round of improvement with the HiCanu assembly, using the same procedure described above. For each diploid genome, the Hifiasm-assembled unitigs were aligned to the DeNovoMAGIC diploid scaffolds with MUMMER4. The reciprocal best alignments with size >5 kb and identity >99.5% were identified. Gap regions in the diploid scaffolds were filled with corresponding sequences of Hifiasm unitigs based on alignments spanning the gaps. Diploid scaffolds aligned to the same unitig with proper order were joined together and the redundant scaffolds (coverage >0.9 and identity >99.5%) were removed. The final diploid scaffolds were used for the second round of improvement with the HiCanu assembly.

### Haplome construction

To construct the two haplomes (haploid genomes) for each accession, we first aligned the diploid assembly against the GDDH13 genome using NUCmer in MUMMER4 (ref. ^65^) with default parameters. Each scaffold of the assembly was assigned to one specific chromosome according to the alignments. All-versus-all BLAST was applied to scaffolds assigned to the same chromosome, and allelic scaffolds that were contained by others were temporarily removed. The remaining scaffolds were aligned to the corresponding chromosome sequences of the GDDH13 genome, and the resulting alignments were filtered by the delta-filter program and subjected to scaffold anchoring using show-tiling in MUMMER4. As the allele redundancy could cause difficulty in scaffold selection at each locus, we used an iterative anchoring approach with manual examination to avoid/minimize the inclusion of (partial) redundant alleles, while keeping the adjacent scaffolds at minimal distance. For each chromosome, we started with the longest scaffold and preserved others that were anchored properly with the longest scaffold. This could take several rounds to include or exclude scaffolds to produce the first haplome (haplome A). Scaffolds not included by haplome A were used for generation of the second haplome (haplome B). Scaffolds assigned to neither haplome A nor haplome B were collectively referred to as unplaced scaffolds. Scaffolds in each of the haplome were used to construct homologous chromosomes with the high-density genetic maps and genomic synteny as described above.

### Repeat annotation and gene prediction

Repeat library for each apple accession was built using EDTA^66^, a de novo transposable element (TE) annotator that integrates structure- and homology-based approaches for TE identification. Protein-coding genes were predicted from repeat-masked assemblies using MAKER-P^67^ (v.2.31.10), which integrates evidence from protein homology, transcripts and ab initio predictions. RNA-seq reads were processed to remove adapters and low-quality bases using Trimmomatic^68^ (v.0.35), and assembled both de novo and genome guided using Trinity^69^ (v.2.4.0). The resulting assembled contigs were used as the transcript evidence. Proteomes of published apple genomes as well as closely related species, including pear, peach and strawberry, were aligned to the assemblies using Exonerate^70^ to provide homology-based evidence. Finally, ab initio gene predictions from SNAP^71^, AUGUSTUS^72^ (v.3.3) and GeneMark-ES^73^ (v.4.35) were integrated with transcript and protein-homology evidence by MAKER-P to generate the final gene models. Protein sequences of the predicted genes were compared against GenBank nonredundant protein (nr) and InterPro databases to identify homology information and protein domains, respectively. Gene Ontology annotation was done using Blast2GO^74^.

### Genetic contribution of wild progenitors to the cultivated apple

A whole genome alignment for each pair of assemblies was performed with NUCmer^65^. To infer genetic contributions of the two wild progenitors to the cultivated apple, we projected all pairwise genome alignments to the Gala haploid consensus genome, and calculated genetic divergence using distmat (http://www.bioinformatics.nl/cgi-bin/emboss/distmat) with the Jukes–Cantor correction for each nonoverlapping 50-kb window on the Gala genome. The window-based genetic divergences were fitted with local regression to show the pattern of relative contribution of the two wild progenitors. To quantify the contributions, we evaluated genetic divergences in all windows with alignment length >100 bp. The mean of the smaller divergences of all windows was 0.8% (± 0.8%), whereas the mean of the larger divergences was 1.4% (± 1.2%). Accordingly, we assigned the genetic origin of each window in the cultivated apple to either *M. sieversii* or *M. sylvestris*, whichever had the smaller divergence value to the cultivated apple genomes, which must be <1.4%. Meantime, we also required that the difference between the two divergence values (*M. sieversii* to the cultivated apple and *M. sylvestris* to the cultivated apple) must be >0.2%, which was the lower bound of the mean difference across all windows (1.3% ± 1.1%). Windows that did not meet the criteria were classified as those with unclear genetic origin. We used variable window sizes (5, 10, 20, 30, 40, 50, 60 and 70 kb) and minimal alignment length cutoffs (100, 300, 500 and 1,000 bp) for the analysis, and obtained similar results.

### Comparative genomics and divergence time estimation

Orthologous groups of selected plant species were constructed using OrthoMCL^75^. Protein sequences of each single-copy orthologous group were aligned with MAFFT^76^ (v.7.313) with parameters ‘--maxiterate 1000 --localpair’. Each alignment was used to build a maximum likelihood phylogeny with IQ-TREE^77^. We then compared each gene tree with the species tree and preserved orthologous groups with gene trees that were consistent with the species tree. Alignments of resulting orthologous groups were concatenated to build a maximum likelihood phylogeny. The divergence time was estimated using MCMCTree^78^ with branch lengths estimated by BASEML in the PAML package^79^ and independent rate model for time estimation. We constrained the root age to <200 Ma and the Rosids age to 128.63 to 85.8 Ma (ref. ^80^), and performed 20,000 samplings with ‘burnin=20000’ and ‘sampfreq=500’.

The apple mutation rate was inferred using the formula *µ* = *D*/2*T*, where *D* is the evolutionary distance of *M. sieversii* and *M. sylvestris* (0.014; Supplementary Fig. 6b) and *T* is the divergence time of the two species (1.8 Ma; Fig. 1b). The mutation rate was estimated to be 3.9 × 10^−9^ substitutions per site per year, which is close to a previous estimation of 4 × 10^−9^ for apples based on a small-scale dataset^19^.

### Variant calling and population genetic analyses

Illumina paired-end reads were processed to remove adapters and low-quality sequences using Trimmomatic^68^. Cleaned reads were mapped to the apple reference genome^23^ using BWA^62^. The resulting alignments were processed with Picard (https://broadinstitute.github.io/picard) to keep those uniquely aligned to the genome and having mapping quality >20. Variants were called using BCFtools (http://samtools.github.io/bcftools) and filtered using VCFtools (http://vcftools.sourceforge.net) with parameters ‘-maf 0.05 -minQ 30 -max-missing 0.9’. A neighbor-joining phylogeny was constructed based on the *P* distance matrix calculated by VCF2Dis (https://github.com/BGI-shenzhen/VCF2Dis), and the principal component analysis was performed with EIGENSOFT^81^. The population structure was analyzed using the STRUCTURE program^82^. The likelihood of *K* from 1 to 11 was estimated using 20,000 randomly selected SNPs. Each *K* was run 20 times and the number of kinships suggesting the most likely number of clusters in the population was obtained. Seven *M. domestica* accessions (A12, R05, R06, R08, R11, R12 and M27) and one *M. sieversii* accession (C84) were excluded from the downstream analyses due to their suspicious taxonomy classifications according to our phylogeny and genomic origin analyses. Genome-wide scan of selective sweeps was performed by comparing allele frequency between *M. domestica* and *M. sieversii* and between *M. domestica* and *M. sylvestris* using XP-CLR with the parameters ‘-w1 0.005 200 1000 -p0 0.95’. Nucleotide diversity (*π*) and Tajima’s *D* were calculated based on a sliding window of 20 kb and a step size of 1 kb. The population-level SVs were identified using LUMPY^83^ and SVs between haplomes were identified using Assemblytics^84^.

### Inference of demographic history

The dynamics of historic population size was inferred using the pairwise sequentially Markovian coalescent (PSMC) model^85^. Reads of selected samples were mapped to the reference genome using BWA^61^, and based on the alignments of heterozygous sites that were identified using SAMtools^86^. The psmc program in the PSMC package was used to estimate the effective population size with parameters ‘-N25 -t15 -r5 -p ‘4 + 25*2 + 4 + 6’’. The estimated apple mutation rate (3.9 × 10^−9^) and the average generation time^19^ (7.5 years) were used to scale the population parameters into years and individuals.

### Pan-genome construction and analysis

To build pan-genomes, cleaned reads of 36 *M. domestica*, 36 *M. sieversii* and 11 *M. sylvesitris* accessions were assembled de novo using SPAdes^87^ (v.3.13.0). The assembled contigs of *M. sieversii* and *M. sylvestris* accessions were aligned to the corresponding reference genomes (haploid consensus assemblies) using QUAST-LG^88^. Contigs containing unaligned segment(s) (identity cutoff 90%) with single-segment length >500 bp were retained. The resulting contigs, probably from nonplant organisms or mitochondrial/chloroplast genomes, were removed. The contigs were further processed with CD-HIT^89^ to remove redundancies with a 90% identity cutoff. For *M. domestica*, first, genes from the four previously published genome assemblies^21–23^ were aligned to the Gala genome to identify high-confidence genes that were not present in Gala (identity <90% or coverage <50%). Then, nonreference sequences of resequencing accessions were identified using the same strategy for *M. sieversii* and *M. sylvestris*, except that contigs were aligned to all of the five *M. domestica* genome assemblies. Protein-coding genes were predicted from the nonredundant unaligned contigs using MAKER-P^67^ as described above.

PAVs of protein-coding genes in the pan-genome for each species were identified using the same method described in Gao et al.^13^. A gene was defined as absent in a given accession when <20% of its coding region was covered by at least two reads. To investigate cross-species PAV patterns, we built orthologue groups of the three pan-genomes. We first used MCScanX^90^ to identify orthologues based on genome synteny. Genes that failed to be classified by MCScanX were grouped using BLASTp following the bidirectional best-hit strategy. Singletons as well as orthologue groups with missing genes in certain species were further checked by aligning their CDS sequences against the genomes using BLAT^91^. For the PAV of an orthologue group in each accession, we defined it as absent in a given accession if none of the orthologues within the group passed the PAV criterion when reads were mapped to the corresponding pan-genomes. The Fisher’s exact test followed by multiple test correction^92^ was used to identify favorable genes in different species (adjusted *P* value <0.05).

### ASE

Cleaned RNA-seq reads were first mapped against both haplomes of Gala using STAR^93^ (v.2.7.3a). Reads uniquely aligned to the same chromosome in both haplomes were preserved and assigned to the allele if the alignment had a higher score and fewer mismatches than to the other allele. Reads that failed to be assigned were mainly from homozygous genomic region or regions that were unassembled or haplotype unresolved. The latter was addressed through SNP analysis. We called heterozygous SNPs in the Gala consensus genome using Illumina paired-end reads with GATK4 (https://gatk.broadinstitute.org). Biallelic SNPs that passed hard filtering using GATK (‘QD < 2, QUAL < 60, SOR > 3, FS > 60, MQ < 40, MQRankSum < −12.5, ReadPosRankSum < −8’) and supported by at least five reads for both reference and alternative alleles were kept, and phased based on the two haplomes. For each SNP site, when genotype information was available for only one haplome, the genotype of another haplome was imputed based on the SNP calls. With the phased SNPs, we further classified the unseparated RNA-seq reads into different alleles using SNPsplit^94^. Reads separated by the above two approaches were combined for downstream ASE analysis. Unlike read assignment biased toward the reference allele in conventional ASE studies, for which only a consensus genome was used as a reference^95^, in the present study the ratio of reads assigned to either haplome was close to 1 (Supplementary Table 13), indicating unbiased read assignment. The allele-specific reads were mapped to the Gala consensus genome using STAR and genes with total counts >10 in all biological replicates were analyzed for ASE using DESeq2 (ref. ^96^). A gene was considered to have ASE if the expression difference of the two alleles was significantly greater than twofold (adjusted *P* < 0.05).

### Reporting Summary

Further information on research design is available in the Nature Research Reporting Summary linked to this article.

## Online content

Any methods, additional references, Nature Research reporting summaries, source data, extended data, supplementary information, acknowledgements, peer review information; details of author contributions and competing interests; and statements of data and code availability are available at 10.1038/s41588-020-00723-9.

## Supplementary information

Supplementary Information Supplementary Note and Supplementary Figs. 1–16.

Reporting Summary

Supplementary Tables Supplementary Tables 1–14.

## Data Availability

Genome assemblies, raw genome and transcriptome sequencing reads have been deposited in the National Center for Biotechnology Information BioProject database (http://www.ncbi.nlm.nih.gov/bioproject) under the accession no. PRJNA591623. Genome assemblies and annotated genes, nonreference genome sequences and annotated genes of the apple pan-genomes, and SNPs and SVs called from the genome resequencing data are also freely available at http://bioinfo.bti.cornell.edu/apple_genome.

## Code availability

Computational pipelines related to assembly validation and improvement can be accessed through https://github.com/XuepengSun/apple_diploid_genomes.

## References

[1] Ross-Ibarra J, Morrell PL, Gaut BS (2007). Plant domestication, a unique opportunity to identify the genetic basis of adaptation. Proc. Natl Acad. Sci. USA.

[2] Shan Q (2013). Targeted genome modification of crop plants using a CRISPR-Cas system. Nat. Biotechnol..

[3] Hickey LT (2019). Breeding crops to feed 10 billion. Nat. Biotechnol..

[4] Soyk S (2016). Variation in the flowering gene SELF PRUNING 5G promotes day-neutrality and early yield in tomato. Nat. Genet..

[5] Tian J (2019). Teosinte ligule allele narrows plant architecture and enhances high-density maize yields. Science.

[6] Brozynska M, Furtado A, Henry RJ (2016). Genomics of crop wild relatives: expanding the gene pool for crop improvement. Plant Biotechnol. J..

[7] Van de Peer Y, Mizrachi E, Marchal K (2017). The evolutionary significance of polyploidy. Nat. Rev. Genet..

[8] Wu S (2018). Genome sequences of two diploid wild relatives of cultivated sweetpotato reveal targets for genetic improvement. Nat. Commun..

[9] Daccord N (2017). High-quality de novo assembly of the apple genome and methylome dynamics of early fruit development. Nat. Genet..

[10] Schnable PS, Springer NM (2013). Progress toward understanding heterosis in crop plants. Annu. Rev. Plant Biol..

[11] Minio A, Massonnet M, Figueroa-Balderas R, Castro A, Cantu D (2019). Diploid genome assembly of the wine grape Carménère. G3.

[12] Zhao Q (2018). Pan-genome analysis highlights the extent of genomic variation in cultivated and wild rice. Nat. Genet..

[13] Gao L (2019). The tomato pan-genome uncovers new genes and a rare allele regulating fruit flavor. Nat. Genet..

[14] Peter J (2018). Genome evolution across 1,011 *Saccharomyces cerevisiae* isolates. Nature.

[15] Rakocevic G (2019). Fast and accurate genomic analyses using genome graphs. Nat. Genet..

[16] Yang X, Lee WP, Ye K, Lee C (2019). One reference genome is not enough. Genome Biol..

[17] Tao Y, Zhao X, Mace E, Henry R, Jordan D (2019). Exploring and exploiting pan-genomics for crop improvement. Mol. Plant.

[18] Cornille A, Giraud T, Smulders MJ, Roldán-Ruiz I, Gladieux P (2014). The domestication and evolutionary ecology of apples. Trends Genet..

[19] Cornille A (2012). New insight into the history of domesticated apple: secondary contribution of the European wild apple to the genome of cultivated varieties. PLoS Genet..

[20] Duan N (2017). Genome re-sequencing reveals the history of apple and supports a two-stage model for fruit enlargement. Nat. Commun..

[21] Li X (2016). Improved hybrid de novo genome assembly of domesticated apple (*Malus × domestica*). GigaScience.

[22] Velasco R (2010). The genome of the domesticated apple (*Malus × domestica* Borkh.). Nat. Genet..

[23] Zhang L (2019). A high-quality apple genome assembly reveals the association of a retrotransposon and red fruit colour. Nat. Commun..

[24] Broggini, G. A. et al. Chromosome-scale *de novo* diploid assembly of the apple cultivar ‘Gala Galaxy’. Preprint at *bioRxiv*10.1101/2020.04.25.058891 (2020).

[25] Chen X (2019). Sequencing of a wild apple (*Malus baccata*) genome unravels the differences between cultivated and wild apple species regarding disease resistance and cold tolerance. G3.

[26] Di Pierro EA (2016). A high-density, multi-parental SNP genetic map on apple validates a new mapping approach for outcrossing species. Hortic. Res..

[27] Howard NP (2017). Elucidation of the ‘Honeycrisp’ pedigree through haplotype analysis with a multi-family integrated SNP linkage map and a large apple (*Malus × domestica*) pedigree-connected SNP data set. Hortic. Res..

[28] Meng D (2018). Sorbitol modulates resistance to *Alternaria alternata* by regulating the expression of an NLR resistance gene in apple. Plant Cell.

[29] Linsmith G (2019). Pseudo-chromosome-length genome assembly of a double haploid ‘Bartlett’ pear (*Pyrus communis* L.). GigaScience.

[30] Chen H, Patterson N, Reich D (2010). Population differentiation as a test for selective sweeps. Genome Res..

[31] Hirakawa Y, Sawa S (2019). Diverse function of plant peptide hormones in local signaling and development. Curr. Opin. Plant Biol..

[32] Rodríguez-Leal D, Lemmon ZH, Man J, Bartlett ME, Lippman ZB (2017). Engineering quantitative trait variation for crop improvement by genome editing. Cell.

[33] Espino-Díaz M, Sepúlveda DR, González-Aguilar G, Olivas GI (2016). Biochemistry of apple aroma: a review. Food Technol. Biotechnol..

[34] Liu Z (2016). Construction of a genetic linkage map and QTL analysis of fruit-related traits in an F1 Red Fuji × Hongrou apple hybrid. Open Life Sci..

[35] Shao A (2017). The auxin biosynthetic *TRYPTOPHAN AMINOTRANSFERASE RELATED TaTAR2.1-3A* increases grain yield of wheat. Plant Physiol..

[36] Bai Y (2012). A natural mutation-led truncation in one of the two aluminum-activated malate transporter-like genes at the *Ma* locus is associated with low fruit acidity in apple. Mol. Genet. Genom..

[37] Li CL (2020). Apple ALMT9 requires a conserved C-terminal domain for malate transport underlying fruit acidity. Plant Physiol..

[38] Longhi S (2013). A candidate gene based approach validates Md-PG1 as the main responsible for a QTL impacting fruit texture in apple (*Malus* × *domestica* Borkh). BMC Plant Biol..

[39] Yeats, T. H. et al. Allelic diversity of NAC18.1 is a major determinant of fruit firmness and harvest date in apple (*Malus domestica*). Preprint at *bioRxiv*10.1101/708040 (2019).

[40] Norelli JL (2017). Genotyping-by-sequencing markers facilitate the identification of quantitative trait loci controlling resistance to *Penicillium expansum* in *Malus sieversii*. PLoS ONE.

[41] Chagné D (2012). QTL and candidate gene mapping for polyphenolic composition in apple fruit. BMC Plant Biol..

[42] Verdu CF (2014). QTL analysis and candidate gene mapping for the polyphenol content in cider apple. PLoS ONE.

[43] Wang L (2018). Genome of wild mandarin and domestication history of mandarin. Mol. Plant.

[44] Zhou Y, Massonnet M, Sanjak JS, Cantu D, Gaut BS (2017). Evolutionary genomics of grape (*Vitis vinifera* ssp. *vinifera*) domestication. Proc. Natl Acad. Sci. USA.

[45] Yu Y (2018). Genome re-sequencing reveals the evolutionary history of peach fruit edibility. Nat. Commun..

[46] Petit J-R (1999). Climate and atmospheric history of the past 420,000 years from the Vostok ice core, Antarctica. Nature.

[47] Clark PU (2009). The last glacial maximum. Science.

[48] Cornille A (2013). Postglacial recolonization history of the European crabapple (*Malus sylvestris* Mill.), a wild contributor to the domesticated apple. Mol. Ecol..

[49] Contreras-Moreira B (2017). Analysis of plant pan-genomes and transcriptomes with GET_HOMOLOGUES-EST, a clustering solution for sequences of the same species. Front. Plant Sci..

[50] Hurgobin B (2018). Homoeologous exchange is a major cause of gene presence/absence variation in the amphidiploid *Brassica napus*. Plant Biotechnol. J..

[51] Golicz AA (2016). The pangenome of an agronomically important crop plant *Brassica oleracea*. Nat. Commun..

[52] Montenegro JD (2017). The pangenome of hexaploid bread wheat. Plant J..

[53] Wang W (2018). Genomic variation in 3,010 diverse accessions of Asian cultivated rice. Nature.

[54] Li Y-h (2014). De novo assembly of soybean wild relatives for pan-genome analysis of diversity and agronomic traits. Nat. Biotechnol..

[55] Gordon SP (2017). Extensive gene content variation in the *Brachypodium distachyon* pan-genome correlates with population structure. Nat. Commun..

[56] Souleyre EJ (2014). The AAT1 locus is critical for the biosynthesis of esters contributing to ‘ripe apple’ flavour in ‘Royal Gala’ and ‘Granny Smith’ apples. Plant J..

[57] Song C (2016). A UDP‐glucosyltransferase functions in both acylphloroglucinol glucoside and anthocyanin biosynthesis in strawberry (*Fragaria* × *ananassa*). Plant J..

[58] Giovannoni J, Nguyen C, Ampofo B, Zhong S, Fei Z (2017). The epigenome and transcriptional dynamics of fruit ripening. Annu. Rev. Plant Biol..

[59] Jacques D, Vandermijnsbrugge K, Lemaire S, Antofie A, Lateur M (2009). Natural distribution and variability of wild apple (*Malus sylvestris*) in Belgium. Belg. J. Bot..

[60] Zhong S (2011). High-throughput Illumina strand-specific RNA sequencing library preparation. Cold Spring Harb. Protoc..

[61] Avni R (2017). Wild emmer genome architecture and diversity elucidate wheat evolution and domestication. Science.

[62] Li H, Durbin R (2009). Fast and accurate short read alignment with Burrows–Wheeler transform. Bioinformatics.

[63] Tang H (2015). ALLMAPS: robust scaffold ordering based on multiple maps. Genome Biol..

[64] Nurk, S. et al. HiCanu: accurate assembly of segmental duplications, satellites, and allelic variants from high-fidelity long reads. *Genome Res.* **30**, 1291–1305 (2020). 10.1101/gr.263566.120 PMC7545148 32801147

[65] Marçais G (2018). MUMmer4: a fast and versatile genome alignment system. PLoS Comput. Biol..

[66] Ou S (2019). Benchmarking transposable element annotation methods for creation of a streamlined, comprehensive pipeline. Genome Biol..

[67] Campbell MS (2014). MAKER-P: a tool kit for the rapid creation, management, and quality control of plant genome annotations. Plant Physiol..

[68] Bolger AM, Lohse M, Usadel B (2014). Trimmomatic: a flexible trimmer for Illumina sequence data. Bioinformatics.

[69] Haas BJ (2013). De novo transcript sequence reconstruction from RNA-seq using the Trinity platform for reference generation and analysis. Nat. Protoc..

[70] Slater GS, Birney E (2005). Automated generation of heuristics for biological sequence comparison. BMC Bioinf..

[71] Korf I (2004). Gene finding in novel genomes. BMC Bioinf..

[72] Stanke M (2006). AUGUSTUS: ab initio prediction of alternative transcripts. Nucleic Acids Res..

[73] Lomsadze A, Ter-Hovhannisyan V, Chernoff YO, Borodovsky M (2005). Gene identification in novel eukaryotic genomes by self-training algorithm. Nucleic Acids Res..

[74] Conesa A (2005). Blast2GO: a universal tool for annotation, visualization and analysis in functional genomics research. Bioinformatics.

[75] Li L, Stoeckert CJ, Roos DS (2003). OrthoMCL: identification of ortholog groups for eukaryotic genomes. Genome Res..

[76] Katoh K, Misawa K, Kuma KI, Miyata T (2002). MAFFT: a novel method for rapid multiple sequence alignment based on fast Fourier transform. Nucleic Acids Res..

[77] Nguyen L-T, Schmidt HA, von Haeseler A, Minh BQ (2014). IQ-TREE: a fast and effective stochastic algorithm for estimating maximum-likelihood phylogenies. Mol. Biol. Evol..

[78] Yang Z, Rannala B (2005). Bayesian estimation of species divergence times under a molecular clock using multiple fossil calibrations with soft bounds. Mol. Biol. Evol..

[79] Yang Z (2007). PAML 4: phylogenetic analysis by maximum likelihood. Mol. Biol. Evol..

[80] Clarke JT, Warnock RC, Donoghue PC (2011). Establishing a time‐scale for plant evolution. N. Phytol..

[81] Patterson N, Price AL, Reich D (2006). Population structure and eigenanalysis. PLoS Genet..

[82] Hubisz MJ, Falush D, Stephens M, Pritchard JK (2009). Inferring weak population structure with the assistance of sample group information. Mol. Ecol. Resour..

[83] Layer RM, Chiang C, Quinlan AR, Hall IM (2014). LUMPY: a probabilistic framework for structural variant discovery. Genome Biol..

[84] Nattestad M, Schatz MC (2016). Assemblytics: a web analytics tool for the detection of variants from an assembly. Bioinformatics.

[85] Li H, Durbin R (2011). Inference of human population history from individual whole-genome sequences. Nature.

[86] Li H (2009). The sequence alignment/map format and SAMtools. Bioinformatics.

[87] Bankevich A (2012). SPAdes: a new genome assembly algorithm and its applications to single-cell sequencing. J. Comput Biol..

[88] Mikheenko A, Prjibelski A, Saveliev V, Antipov D, Gurevich A (2018). Versatile genome assembly evaluation with QUAST-LG. Bioinformatics.

[89] Fu L, Niu B, Zhu Z, Wu S, Li W (2012). CD-HIT: accelerated for clustering the next-generation sequencing data. Bioinformatics.

[90] Wang Y (2012). MCScanX: a toolkit for detection and evolutionary analysis of gene synteny and collinearity. Nucleic Acids Res..

[91] Kent WJ (2002). BLAT—the BLAST-like alignment tool. Genome Res.

[92] Benjamini Y, Hochberg Y (1995). Controlling the false discovery rate: a practical and powerful approach to multiple testing. J. R. Stat. Soc. Ser. B.

[93] Dobin A (2013). STAR: ultrafast universal RNA-seq aligner. Bioinformatics.

[94] Krueger, F. & Andrews, S. R. SNPsplit: allele-specific splitting of alignments between genomes with known SNP genotypes. *F1000 Research* **5**, 1479 (2016). 10.12688/f1000research.9037.1 PMC4934512 27429743

[95] Castel SE, Levy-Moonshine A, Mohammadi P, Banks E, Lappalainen T (2015). Tools and best practices for data processing in allelic expression analysis. Genome Biol..

[96] Love MI, Huber W, Anders S (2014). Moderated estimation of fold change and dispersion for RNA-seq data with DESeq2. Genome Biol..

