# Supplementary material for: Phased diploid genome assemblies and pan-genomes provide insights into the genetic history of apple domestication
Source: Nat Genet. 2020 Nov 2;52(12):1423–32. doi: 10.1038/s41588-020-00723-9 (PMC7728601; doi:10.1038/s41588-020-00723-9)
Supplement: Supplementary file 1 — Supplementary Note and Supplementary Figs. 1–16. [file 41588_2020_723_MOESM1_ESM.pdf]

nature research

https://doi.org/10.1038/s41588-020-00723-9

Supplementary information

Phased diploid genome assemblies and pan-genomes provide insights into the genetic history of apple domestication

In the format provided by the authors and unedited

Phased diploid genome assemblies and pan-genomes provide insights into the genetic history of apple domestication

Xuepeng Sun[1,8], Chen Jiao[1,8], Heidi Schwaninger[2,8,9], C. Thomas Chao[2], Yumin Ma[3], Naibin Duan[3], Awais Khan[4], Seunghyun Ban[5], Kenong Xu[5], Lailiang Cheng[6], Gan-Yuan Zhong[2,*], Zhangjun Fei[1,7,*]

[1]Boyce Thompson Institute for Plant Research, Cornell University, Ithaca, NY, USA

[2]U.S. Department of Agriculture-Agricultural Research Service, Plant Genetic Resources Unit, Geneva, NY, USA

[3]Shandong Centre of Crop Germplasm Resources, Shandong Academy of Agricultural Sciences, Jinan, Shandong, P.R. China

[4]Plant Pathology and Plant-Microbe Biology Section, School of Integrative Plant Science, Cornell University, Geneva, NY, USA

[5]Horticulture Section, School of Integrative Plant Science, New York State Agricultural Experiment Station, Cornell University, Geneva, NY, USA

[6]Horticulture Section, School of Integrative Plant Science, Cornell University, Ithaca, NY, USA

[7]U.S. Department of Agriculture-Agricultural Research Service, Robert W. Holley Center for Agriculture and Health, Ithaca, NY, USA

[8]These authors contributed equally

[9]Deceased

*email: zf25@cornell.edu; GanYuan.Zhong@ars.usda.gov

Contents

Supplementary Note *3*

Evaluation of apple genome assemblies *3*

1) *Mapping of DNA reads against the assembly* 3
2) *BUSCO evaluation* 3
3) *Whole genome alignment and collinearity with genetic maps* 3
4) *LTR assembly index* 4
5) *K-mer spectrum analysis* 4

Disease resistance gene *4*

LTR-RT bursts and their contribution to apple evolution *5*

Reference *6*

Supplementary Figures *8*

Supplementary Note

Evaluation of apple genome assemblies

We used multiple approaches to evaluate the quality of the apple genome assemblies. All analyses generated competitive metrics compared to the recently published high-quality assemblies of the double-haploid GDDH13 (ref. [1]) and the triple-haploid HFTH1 (ref. [2]).

1) Mapping of DNA reads against the assembly

We used reads from the paired-end (PE) libraries with an insert size of 470 bp from this study and randomly selected PE libraries from previous studies[1,2] to evaluate the genome assemblies based on read mapping rates. For the haploid consensuses, the mapping rates were 99.38% (properly paired: 90.00%), 99.40% (properly paired: 91.75%) and 99.50% (properly paired: 94.54%) for Gala, *M. sieversii* and *M. sylvestris*, respectively. As expected, the mapping rates to the diploid assemblies were higher, particularly the rates of properly mapped read pairs, 99.73% (mapped) and 94.39% (properly paired) for Gala, 99.53% and 92.97% for *M. sieversii*, and 99.75% and 97.86% for *M. sylvestris*. As a comparison, the mapping rates to GDDH13 and HFTH1 genome assemblies were 92.64% (properly paired: 86.33%) and 98.50% (properly paired: 96.58%), respectively.

2) BUSCO evaluation

We performed BUSCO[3] analysis on the assemblies. The percentage of complete BUSCO captured by the Gala (haploid: 97.9%; diploid: 97.7%), *M. sieversii* (haploid: 97.9%; diploid: 97.7%) and *M. sylvestris* (haploid: 97.9%; diploid: 97.7%) genome assemblies were high and comparable to that of GDDH13 (97.4%) and HFTH1 (98.2%) (**Supplementary Table 2**).

3) Whole genome alignment and collinearity with genetic maps

Whole genome alignments between our assemblies and GDDH13 (**Supplementary Fig. 3**) showed good collinearity between all of these assemblies. Furthermore, the genome assemblies also had high collinearity with the two recently published apple genetic maps[4,5]. Together these results suggested that our assemblies and chromosome anchoring are of high quality.

4) LTR assembly index

The LTR assembly index (LAI) provides a reference-free genome contiguity evaluation based on LTR-RTs[6]. The LAI value positively correlates with the quality of assembly, and is usually larger than 10 in reference-quality assemblies. LAI values for Gala, *M. sieversii* and *M. sylvestris* genome assemblies were 14.79, 17.41 and 18.32, respectively, which were comparable to the values of GDDH13 (17.53) and HFTH1 (19.60).

5) K-mer spectrum analysis

The 27-mer spectrum analysis, which compared the diversity and abundance of all distinctive 27-mers in the PE libraries and the assembled genomes, indicated that our diploid assemblies successfully captured most of the genome contents (94-98% of 27-mers) present in the PE libraries (**Extended Data Fig. 1**). A considerable amount of 27-mers (9-13%) were missing in the haploid consensus assemblies, and these 27-mers were presumably encoded by alternative alleles. This is expected as lacking of some *k*-mers is a common feature for a haploid consensus assembled from a heterozygous genome.

Disease resistance gene

Improving disease resistance is one of the main goals in current apple breeding programs. Most plant disease resistance genes encode nucleotide-binding site leucine-rich repeat (NLR) proteins. We identified 170 to 562 NLR proteins in the genomes of three cultivated apples, Gala, GDDH13 and HFTH1, and their two wild progenitors, *M. sieversii* and *M. sylvestris* (**Supplementary Table 3**). The HFTH1 genome encodes an exceptionally low number of NLR proteins (170) compared to that of GDDH13 (514) and Gala (562). We found that 373 NLR proteins in GDDH13 could be identified in the HFTH1 genome under the stringent criteria (identity > 95% & coverage > 80%), suggesting that a majority of NLR genes could be mis-annotated in HFTH1, which agrees with the finding that NLR gene prediction is sensitive to annotation pipelines[7]. NLR genes are often clustered in the genome and disease resistance sometimes requires joint action of two adjacent NLR genes with the head-to-head configuration[8,9]. We identified 34-112 NLR gene clusters in different assemblies, which accounted for 65-77% of total NLR genes (**Supplementary Table 3**). Approximately 32-55% of adjacent NLR gene pairs were heterogeneous (encoding different domains) and 4-16% were arranged head-to-head, suggesting that they were not simply derived

from local duplications. The chromosomal distribution of NLR genes/clusters was not uniform, with the highest density on chromosome 2 (**Supplementary Fig 2**). Among chromosome pairs arising from whole genome duplication[10], chromosomes 3 and 11 harbored significantly different number of NLR genes, which is likely due to an expansion of NLR genes/clusters on chromosome 11 (**Supplementary Fig 2**). The difference of NLR genes/clusters between the varieties and species was obvious on some chromosomes. Given that many NLR genes/clusters approximate to or overlap with disease resistance QTLs[11-13], the expansion of NLR genes on particular chromosomes might be a consequence of adaptive evolution, which can provide selective advantage during apple evolution and domestication.

LTR-RT bursts and their contribution to apple evolution

We identified 13,196, 15,873 and 14,246 intact LTR-RTs in the diploid genomes of Gala, *M. sieversii* and *M. sylvestris*, respectively. Insertion time estimation of these LTR-RTs unraveled two bursts that occurred at the same periods in all three accessions. The older burst took place 5.69-5.50 million years ago (mya), which predated the divergence of apple and pear (5.1 mya; **Fig. 1b,c** and **Supplementary Fig. 6**). Concordantly, a similar but weaker peak was found in pear (**Supplementary Fig. 7**). The second LTR-RT burst occurred 1.17-1.07 mya, prior to the time when *M. sylvestris* and *M. sieversii* were diversified into subpopulations, respectively (**Fig. 1b,c**). Transposable element (TE) insertions are often deleterious, and therefore are subjective to purifying selection[14,15]. However, the occurrence of whole genome duplication (WGD) in the common ancestor of apple and pear may have provided a relaxed environment for LTR-RT proliferation as increased gene dosage is presumably more tolerant to the deleterious effects of TE transposition. It is worth noting that TE burst is not necessarily accompanied by WGDs as other mechanisms (e.g. horizontal transfer) can also contribute to TE family expansion in different systems.

Historical and recent TE proliferation has changed the diversity and abundance of TE families across species. Consequently, 56-57% of the LTR-RT insertions in the *M. sieversii* or the *M. sylvestris* genome were not found in their syntenic regions. Similarly, 39-51% of the LTR-RT insertions in the genomes of wild species were not found in cultivated apples, suggesting that only partial LTR-RT diversity present in the wild progenitors was inherited by cultivated apples (**Extended Data Fig. 4a**), which as a result inflated genetic diversity among apple varieties

(**Extended Data Fig. 4b**). Inheritance of adaptive LTR-RTs from different progenitors may have a profound impact on fruit traits. One such example is the redTE LTR-RT, whose insertion in the upstream region of *MYB1*, a transcription factor known to control fruit skin color[16], enhanced the *MYB1* expression, leading to red skin color[1]. We found that redTE was present in the *M. sieversii* genome but not in the upstream of *MYB1*, while the *M. sylvestris* genome did not harbor redTE. The red-skinned HFTH1 inherited the *MYB1* locus from *M. sieversii* and underwent a recent redTE transposition into the upstream of *MYB1*. The yellow-skinned GDDH13 inherited the *MYB1* locus from *M. sylvestris*, and likely passed it to Gala, as it is one of the parents of Gala. Interestingly, Gala has another *MYB1* allele that originated from *M. sieversii* with a different LTR-RT insertion in the upstream of *MYB1*. We found that this insertion was originated from redTE but only left with the LTR sequences (solo-LTR) surrounded by target site duplications, likely derived from homologous recombination of redTE (**Extended Data Fig. 4c**). Since the solo-LTR itself encodes functional elements and is sufficient to enhance *MYB1* expression[1], we hypothesize that the allele-specific expression of *MYB1* caused by redTE could have contributed to fruit skin color of Gala (**Extended Data Fig. 4c**). Unfortunately, the CDS and UTR sequences of the two *MYB1* alleles in Gala are identical, which prevented us from explicitly investigating the effect of redTE on the allele-specific expression of *MYB1* using the RNA-Seq data we generated.

Reference

1. Zhang, L. *et al.* A high-quality apple genome assembly reveals the association of a retrotransposon and red fruit colour. *Nat. Commun.* **10**, 1494 (2019).
2. Daccord, N. *et al.* High-quality de novo assembly of the apple genome and methylome dynamics of early fruit development. *Nat. Genet.* **49**, 1099–1106 (2017).
3. Simão, F.A., Waterhouse, R.M., Ioannidis, P., Kriventseva, E.V. & Zdobnov, E.M. BUSCO: assessing genome assembly and annotation completeness with single-copy orthologs. *Bioinformatics* **31**, 3210–3212 (2015).
4. Di Pierro, E.A. *et al.* A high-density, multi-parental SNP genetic map on apple validates a new mapping approach for outcrossing species. *Hortic. Res.* **3**, 16057 (2016).
5. Howard, N.P. *et al.* Elucidation of the 'Honeycrisp' pedigree through haplotype analysis with a multi-family integrated SNP linkage map and a large apple (*Malus* x *domestica*) pedigree-connected SNP data set. *Hortic Res* **4**, 17003 (2017).
6. Ou, S., Chen, J. & Jiang, N. Assessing genome assembly quality using the LTR Assembly Index (LAI). *Nucleic Acids Res.* **46**, e126-e126 (2018).
7. Bayer, P.E., Edwards, D. & Batley, J. Bias in resistance gene prediction due to repeat masking. *Nat. Plants* **4**, 762–765 (2018).
8. Okuyama, Y. *et al.* A multifaceted genomics approach allows the isolation of the rice Pia-blast resistance gene consisting of two adjacent NBS-LRR protein genes. *Plant J.* **66**, 467–479 (2011).
9. Bernoux, M., Moncuquet, P., Kroj, T. & Dodds, P.N. A novel conserved mechanism for plant NLR protein pairs: the 'integrated decoy' hypothesis. *Front. Plant Sci.* **5**, 606 (2014).

10. Velasco, R. *et al.* The genome of the domesticated apple (*Malus* x *domestica* Borkh.). *Nat. Genet.* **42**, 833–839 (2010).
11. Durel, C.-E., Denancé, C. & Brisset, M.-N. Two distinct major QTL for resistance to fire blight co-localize on linkage group 12 in apple genotypes 'Evereste' and *Malus floribunda* clone 821. *Genome* **52**, 139–147 (2009).
12. Peil, A. *et al.* Strong evidence for a fire blight resistance gene of *Malus robusta* located on linkage group 3. *Plant Breed.* **126**, 470–475 (2007).
13. Khan, M.A., Zhao, Y. & Korban, S.S. Identification of genetic loci associated with fire blight resistance in *Malus* through combined use of QTL and association mapping. *Physiol. Plant.* **148**, 344–353 (2013).
14. Jangam, D., Feschotte, C. & Betran, E. Transposable element domestication as an adaptation to evolutionary conflicts. *Trends Genet.* **33**, 817–831 (2017).
15. Peng, Y. *et al.* Elimination of a retrotransposon for quenching genome instability in modern rice. *Mol. Plant* **12**, 1395–1407 (2019).
16. Allan, A.C., Hellens, R.P. & Laing, W.A. MYB transcription factors that colour our fruit. *Trends Plant Sci.* **13**, 99–102 (2008).

Supplementary Figures

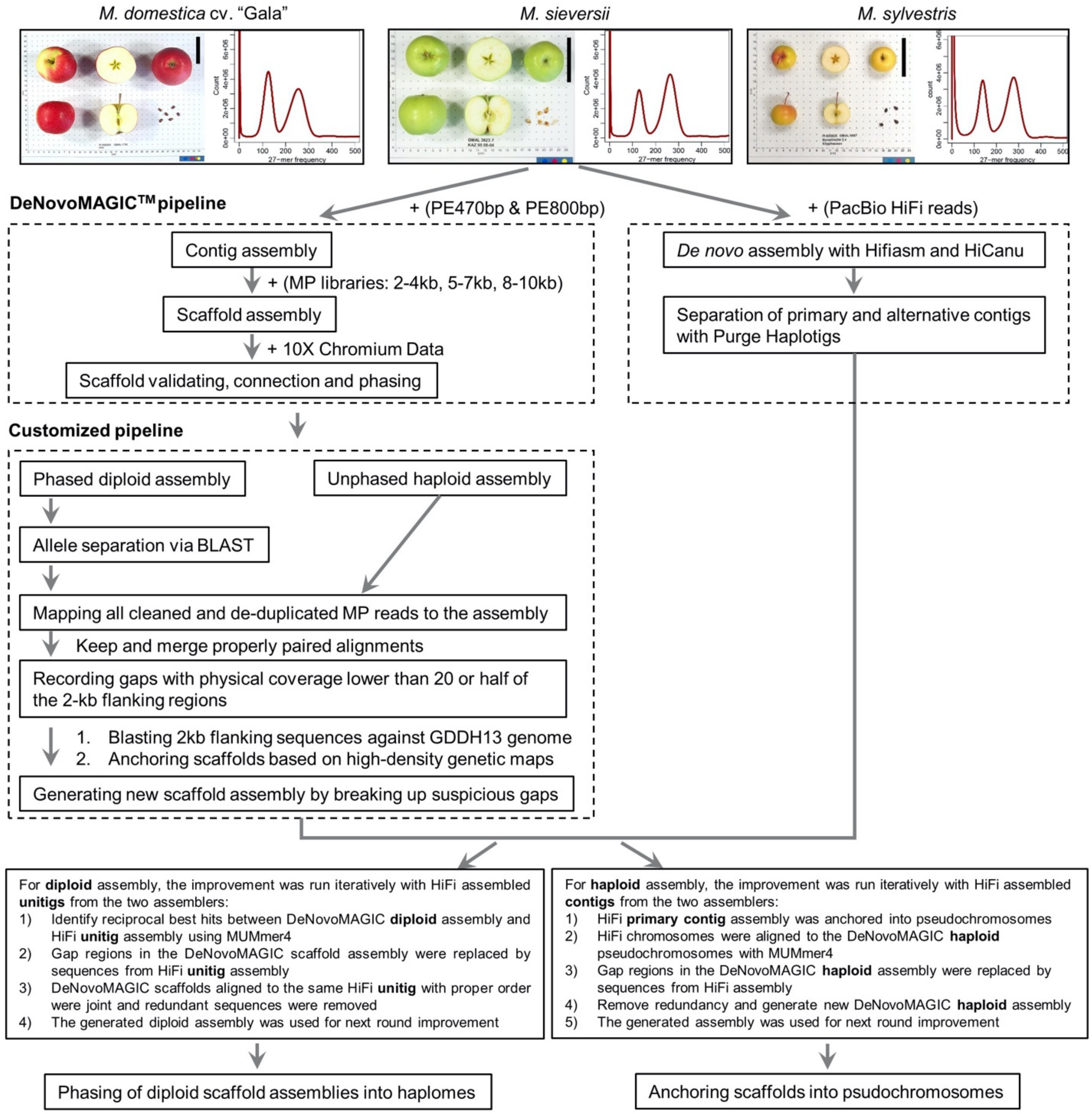

Supplementary Fig. 1 Workflow for genome assembly, error correction, phasing and anchoring. Pictures representing the apple accessions were retrieved from the GRIN database (https://www.ars-grin.gov/) and the heterozygosity was estimated based on the *k*-mer distribution of reads from paired-end libraries (right figure of each panel on the top). Scale bars represent 5 cm.

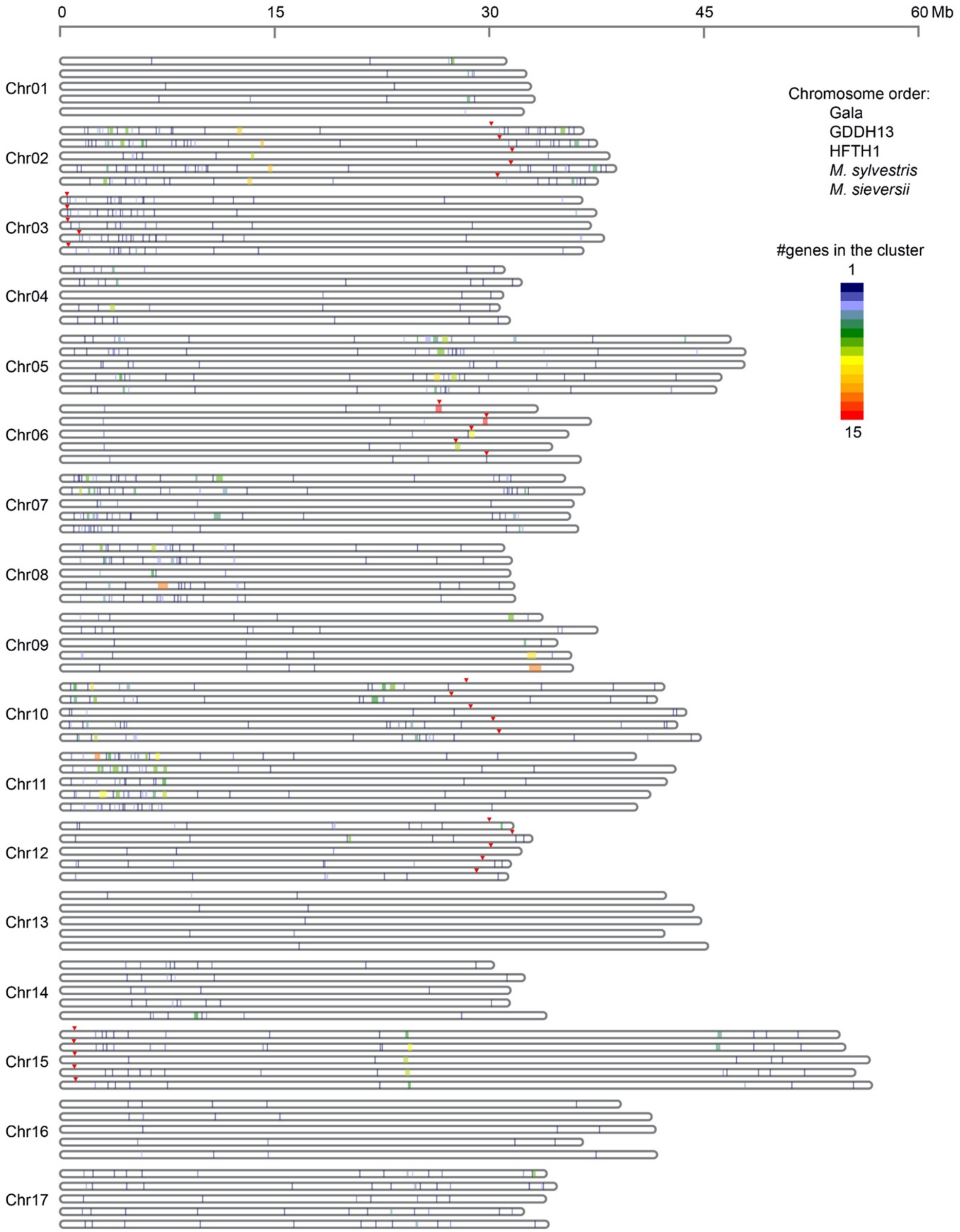

Supplementary Fig. 2 NLR gene clusters in the five apple genome assemblies. The NLR gene clusters and singletons are plotted on each chromosome. For each chromosome, genomes are ordered as follows: Gala, GDDH13, HFTH1, *M. sylvestris* and *M. sieversii*. The size of gene clusters is indicated by the color and band width. Red triangles indicate fire blight resistance QTLs.

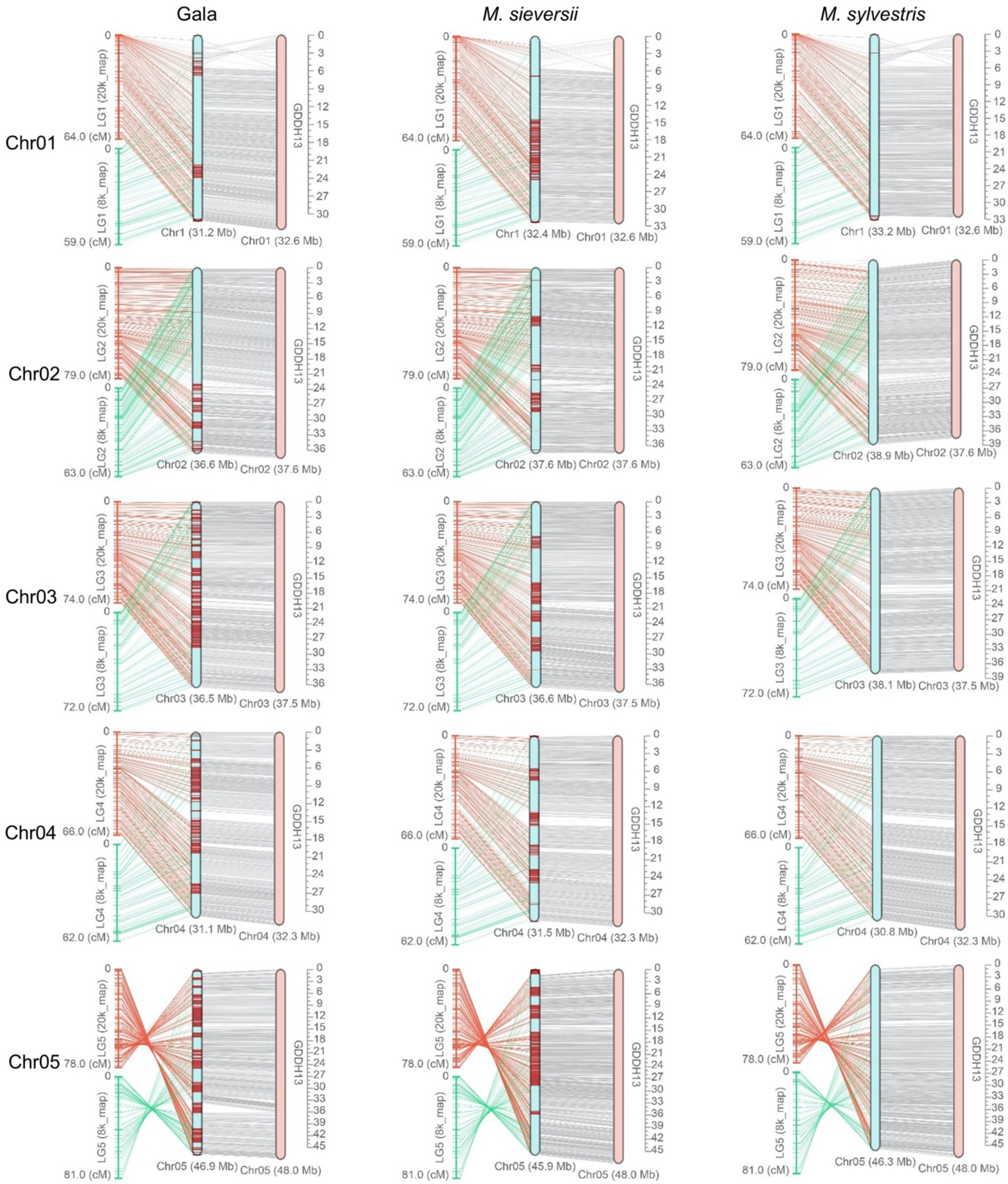

Supplementary Fig. 3 Collinearity between pseudo-chromosomes and genetic maps, and the GDDH13 genome. Two high-density genetic maps are shown on the left and genomic synteny between the pseudo-chromosome (the middle panel) and the GDDH13 genome is shown on the right.

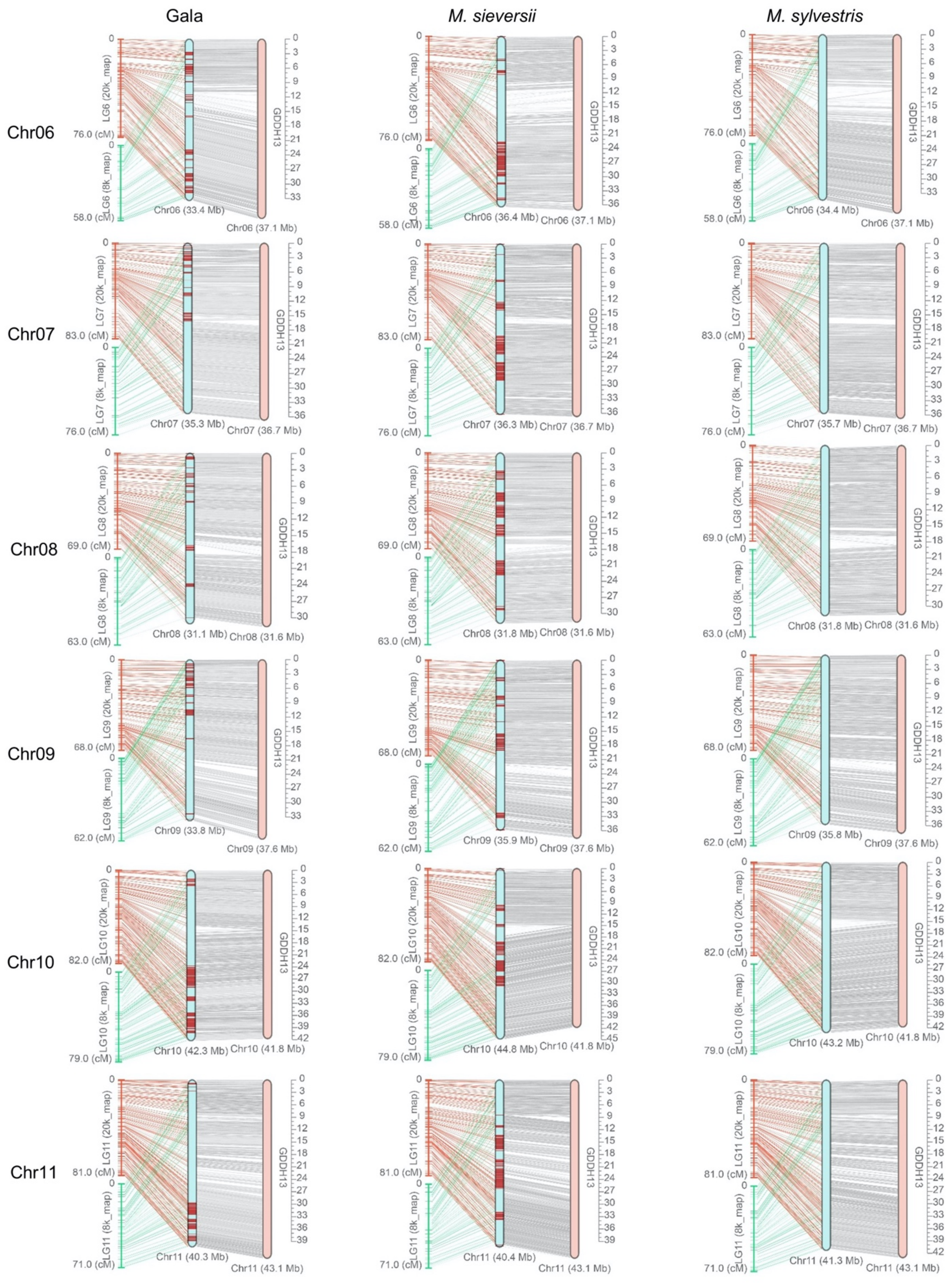

Supplementary Fig. 3 (Continued)

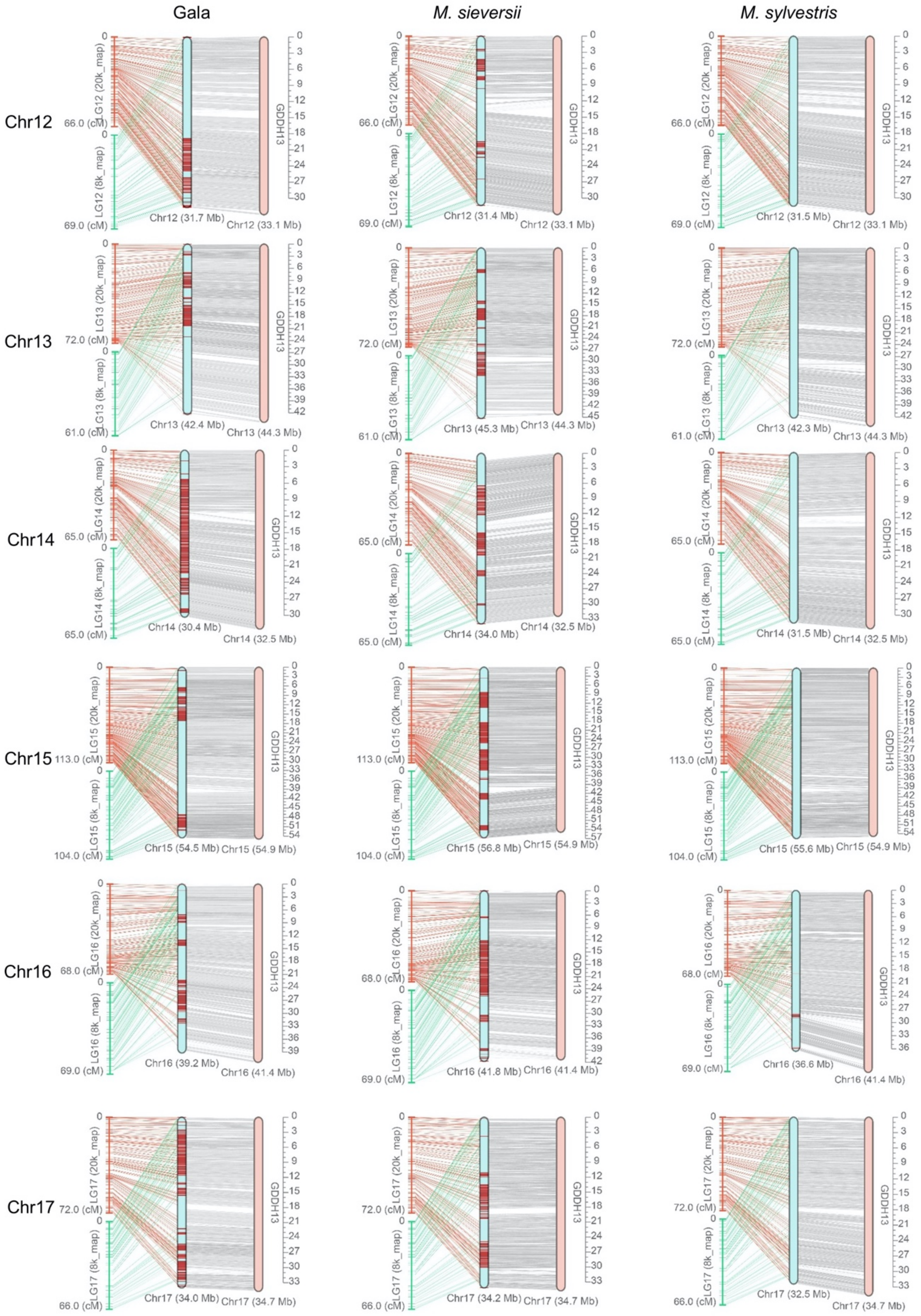

Supplementary Fig. 3 (Continued)

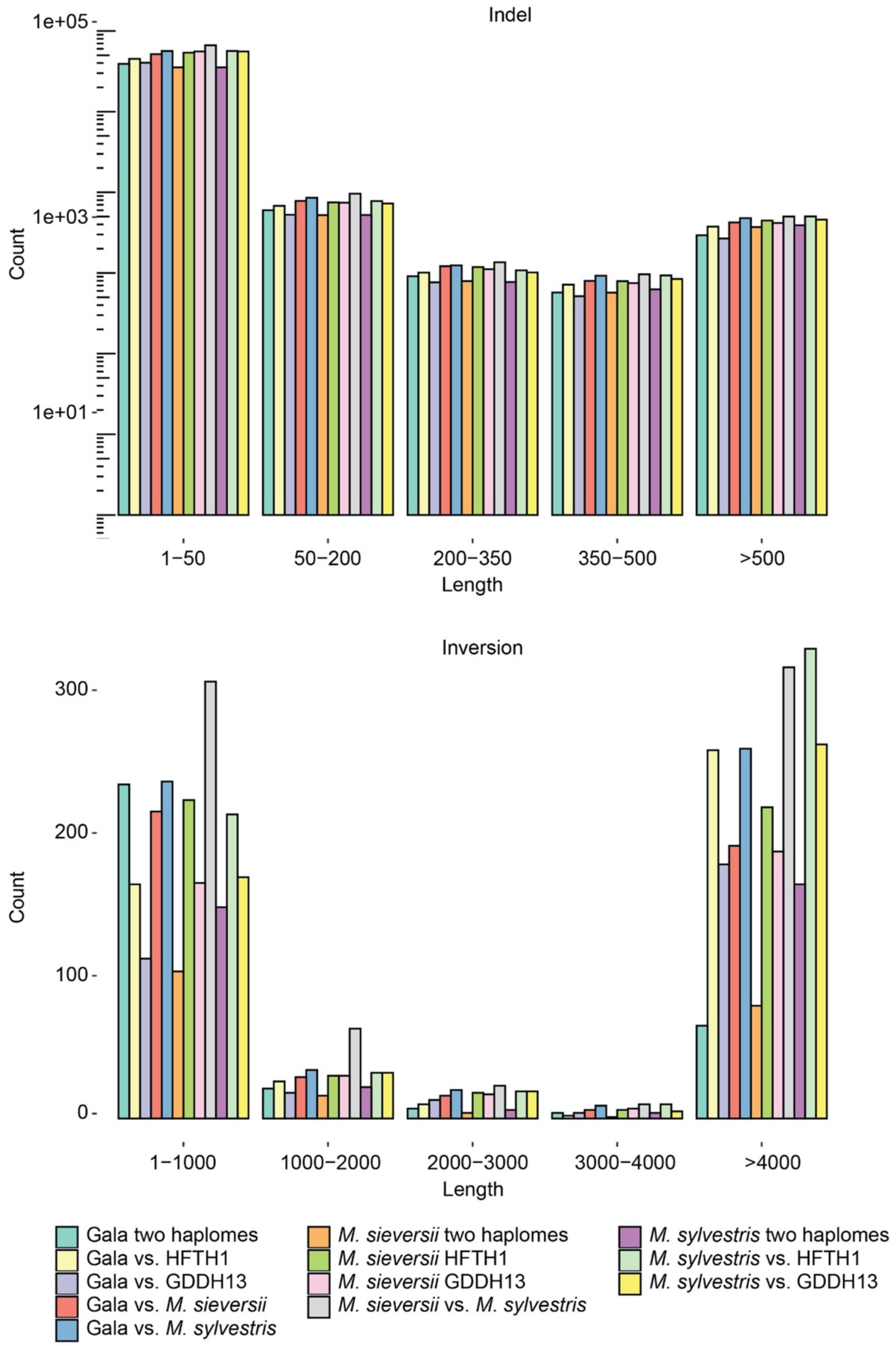

Supplementary Fig. 4 Size distribution of structure variants between different assemblies and between haplomes.

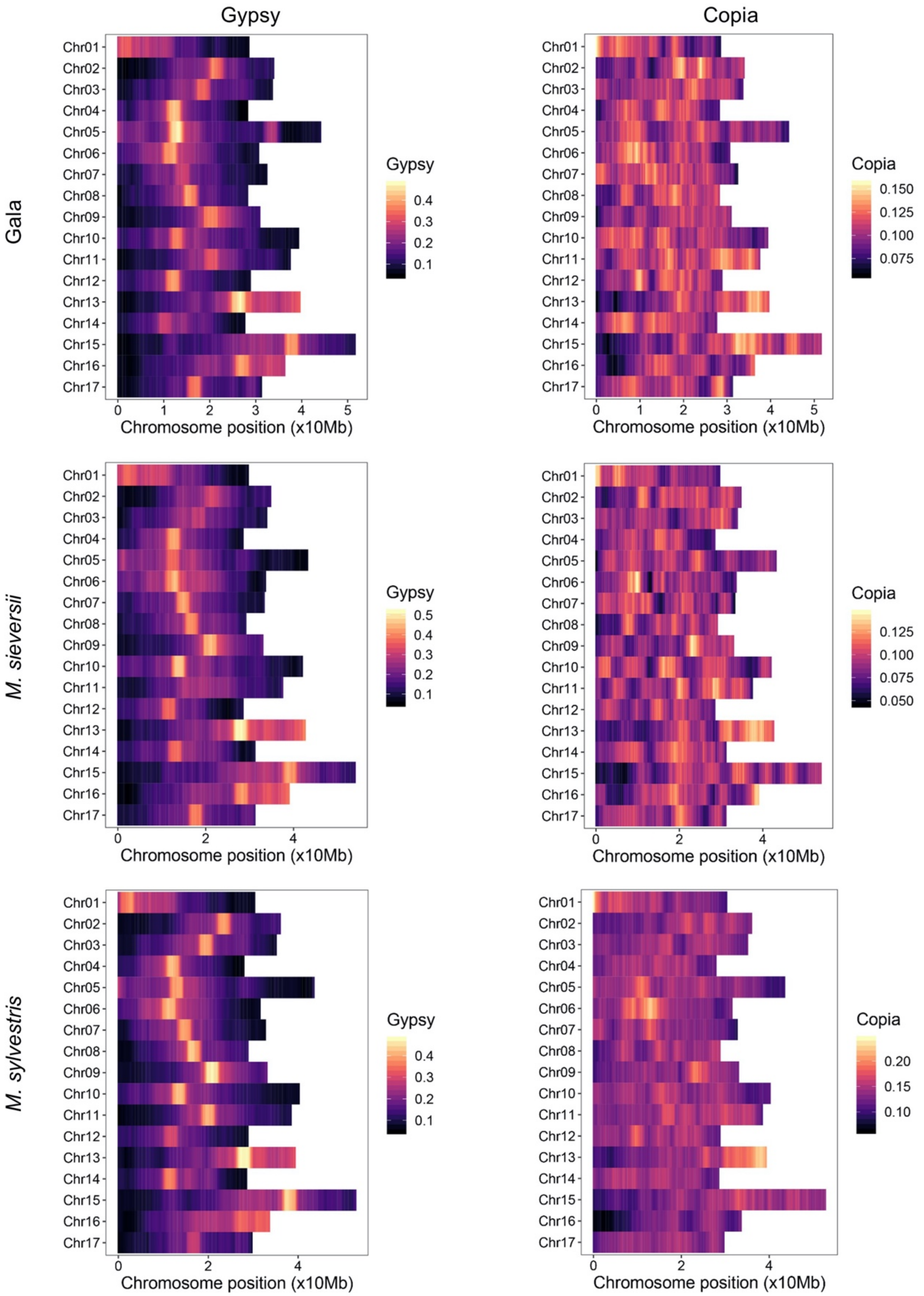

Supplementary Fig. 5 Fraction and distribution of *gypsy* and *copia* retrotransposons in the genomes of the three apple accessions. The fraction was calculated based on a sliding window of 3 Mb and a step size of 300 kb.

a

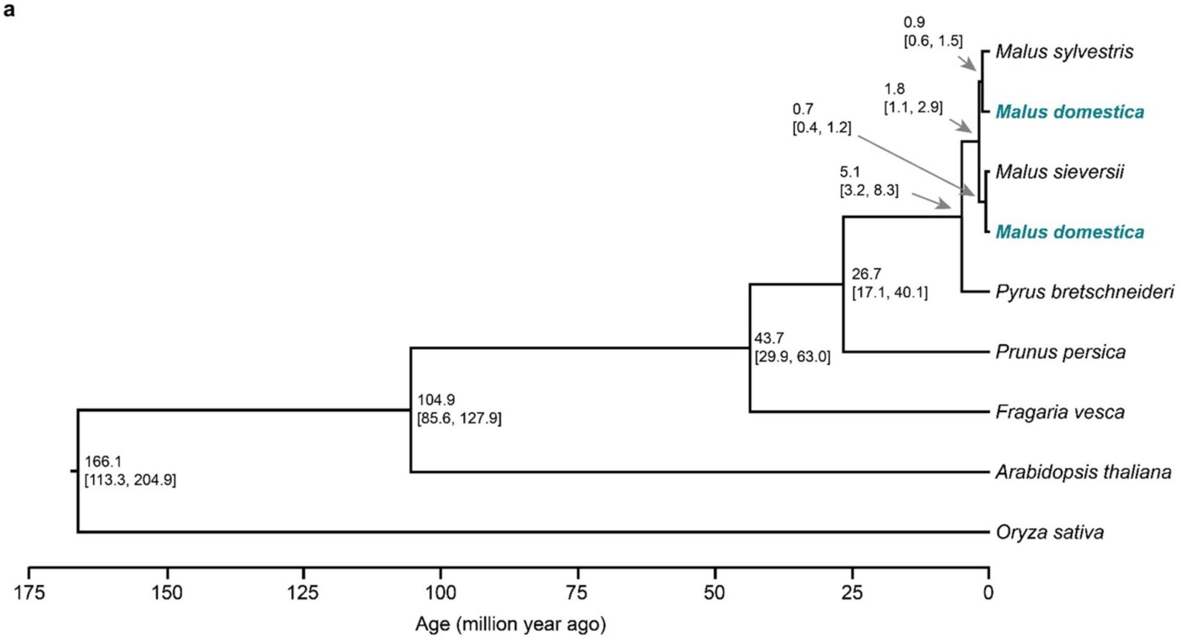

b

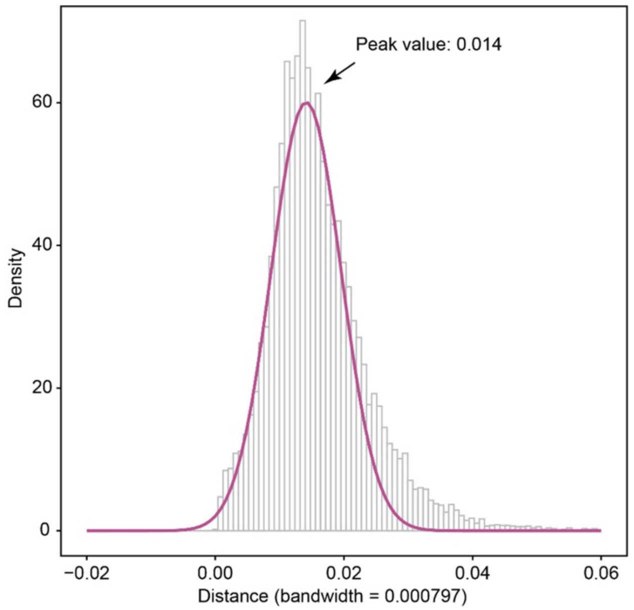

Supplementary Fig. 6 Divergence time and evolutionary distance of the *Malus* species. **a**, Divergence time estimation based on 481 single-copy orthologous groups (OGs). *M. domestica* genes in these OGs clustered either with *M. sieversii* (256 OGs) or with *M. sylvestris* (225 OGs); therefore, these OGs were used to infer the divergence time of *M. sieversii* subpopulations (extant population vs *M. domestica* direct progenitor population) and *M. sylvestris* subpopulations separately. The tree was constrained with a Rosids age between 128.63-85.8 mya and a root age < 200 mya. **b**, Histogram and Gaussian modeling of evolutionary distance between *M. sieversii* and *M. sylvestris*.

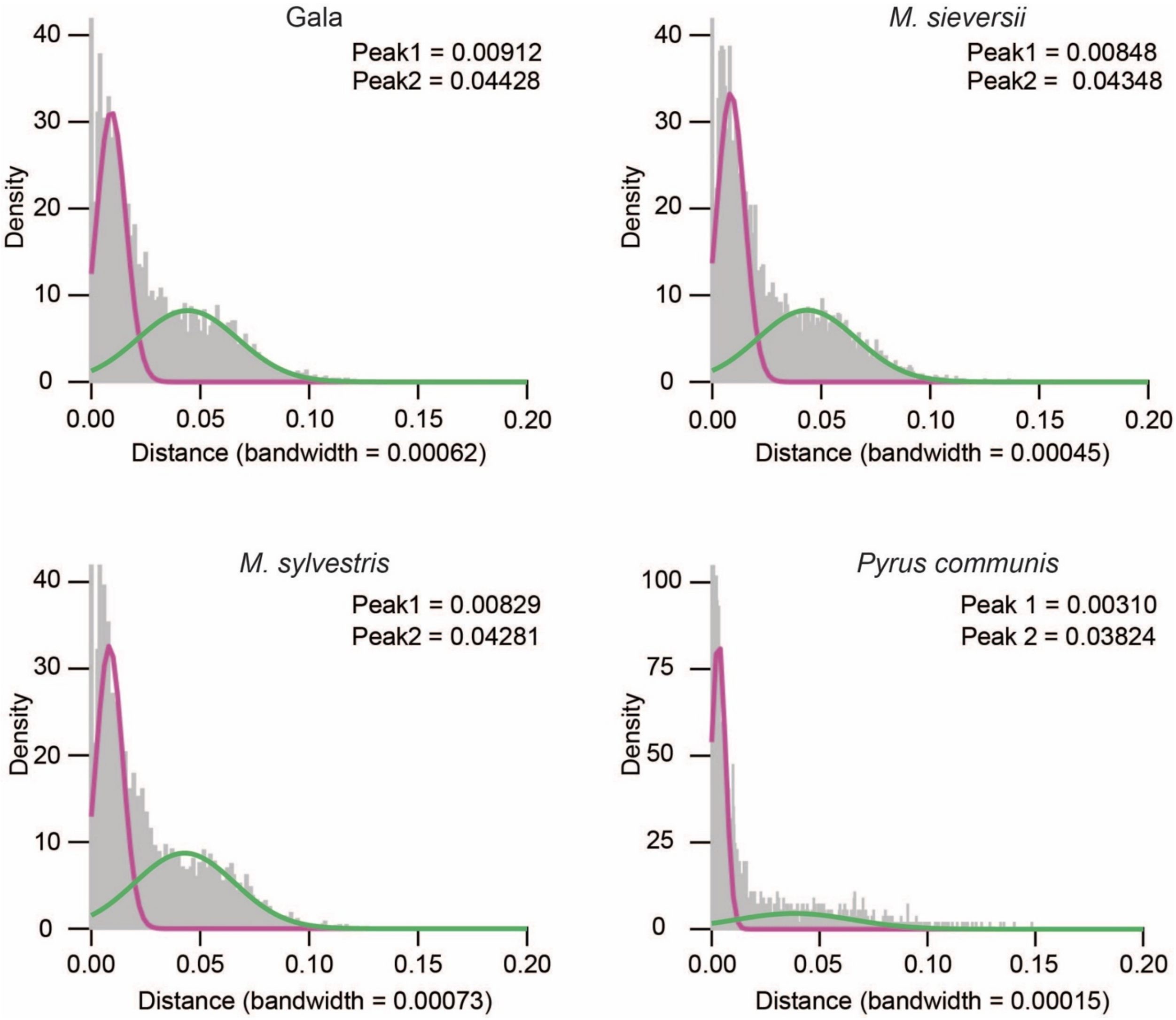

Supplementary Fig. 7 Distribution of LTR insertion time as measured by the evolutionary distance of LTR sequences. The histogram was fitted with the Gaussian mixture model (only the part with positive distance values was plotted). The first two components were plotted and peak value of each component was shown. A total of 13,196, 15,873, 14,264 and 3,580 intact LTR-RTs from Gala, *Malus sieversii*, *M. sylvestris*, and *Pyrus communis*, respectively, were used for the analysis.

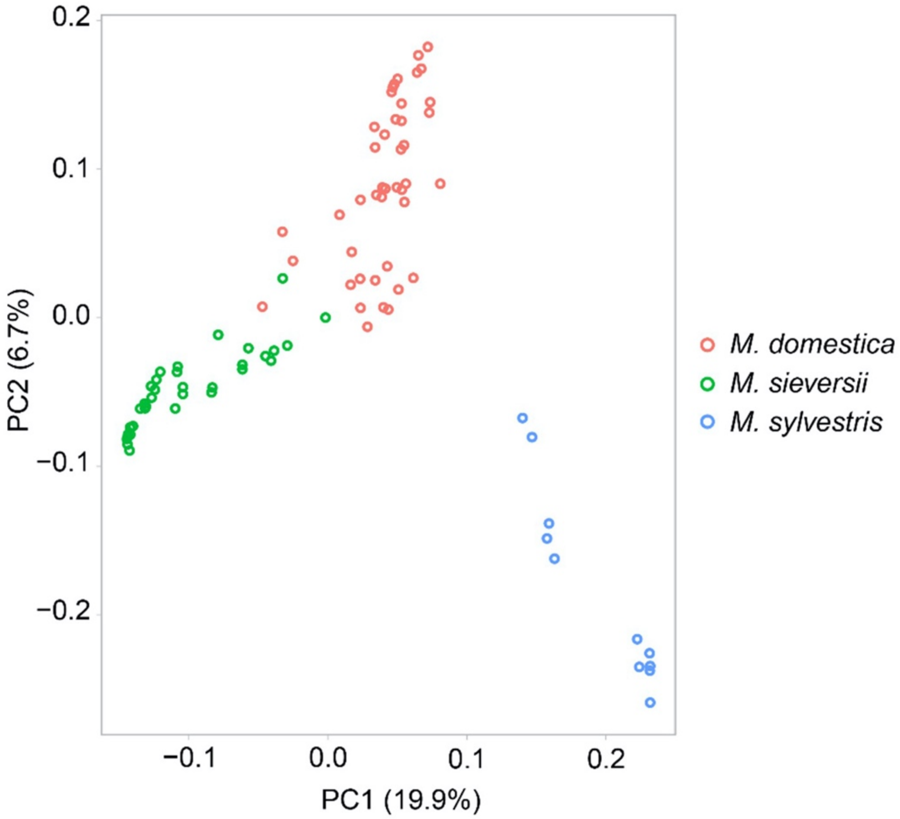

Supplementary Fig. 8 Principal component analysis of 91 *Malus* accessions using 9,988,777 bi-allelic SNPs.

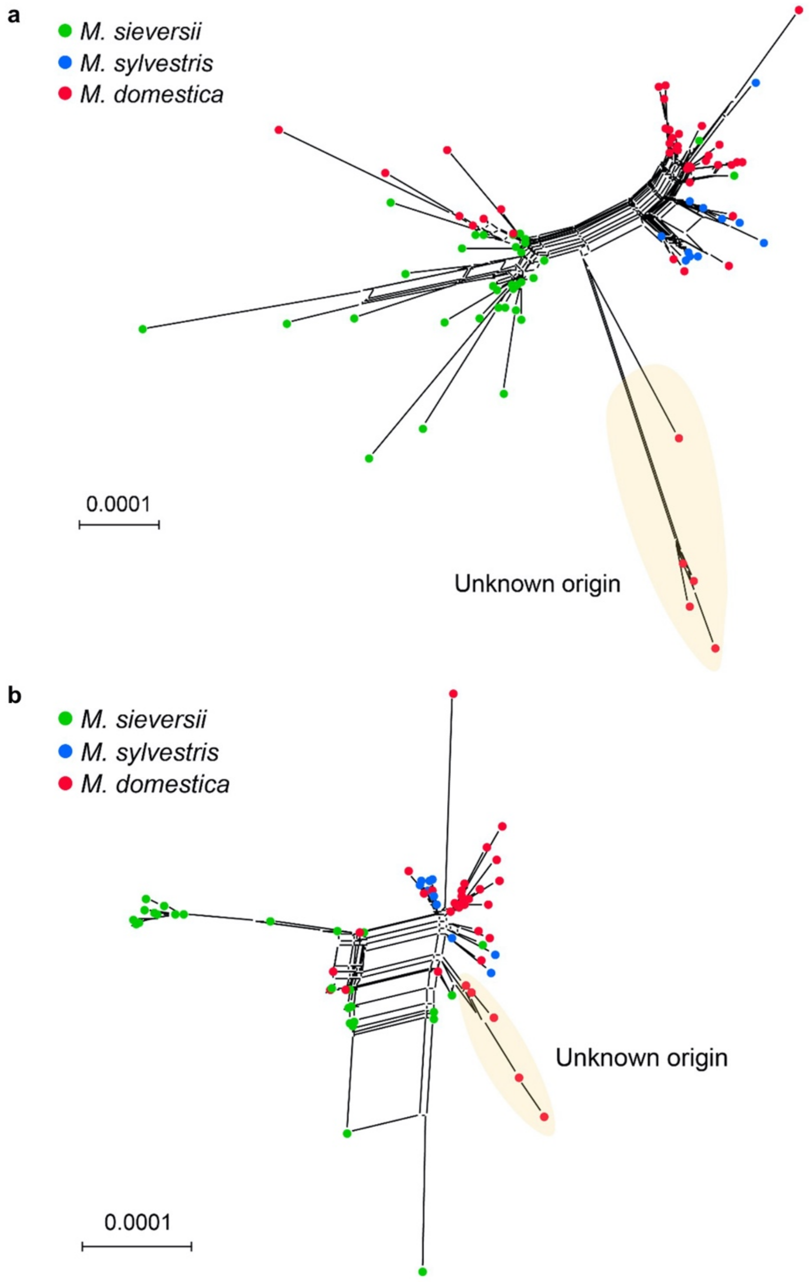

Supplementary Fig. 9 Split networks showing the relative genetic distance of organelle genomes of different *Malus* accessions. The complete genome sequences of mitochondrion (**a**) or chloroplast (**b**) were aligned using clustal omega (https://www.ebi.ac.uk/Tools/msa/clustalo/). Split networks were inferred and visualized with SplitsTree4 (https://github.com/husonlab/splitstree4) based on the whole genome alignments. Some accessions (e.g. R05, R06, R08, R11, and M27) showed unusual genomic composition, which was consistent with their phylogenetic placement based on nuclear genome SNPs. Given that many of these accessions are rootstocks, this suggests that they may not belong to *M. domestica*, or otherwise they may have undergone substantial genetic introgression from other *Malus* species.

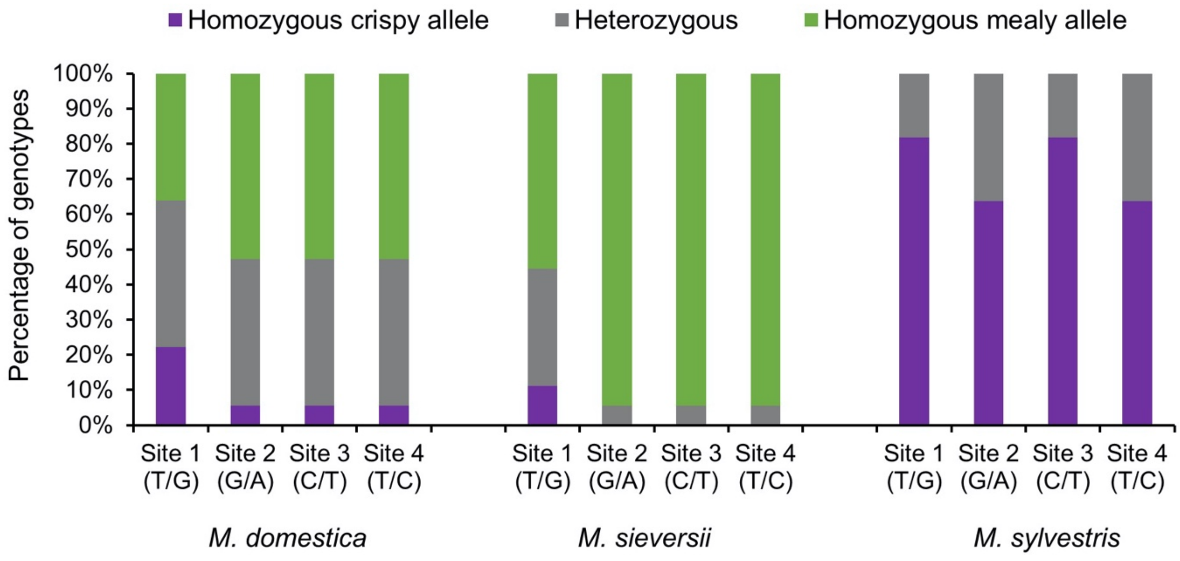

Supplementary Fig. 10 Percentage of the *PG1* genotypes comprising alleles associated with mealy or crispy texture of apple fruit in three *Malus* populations.

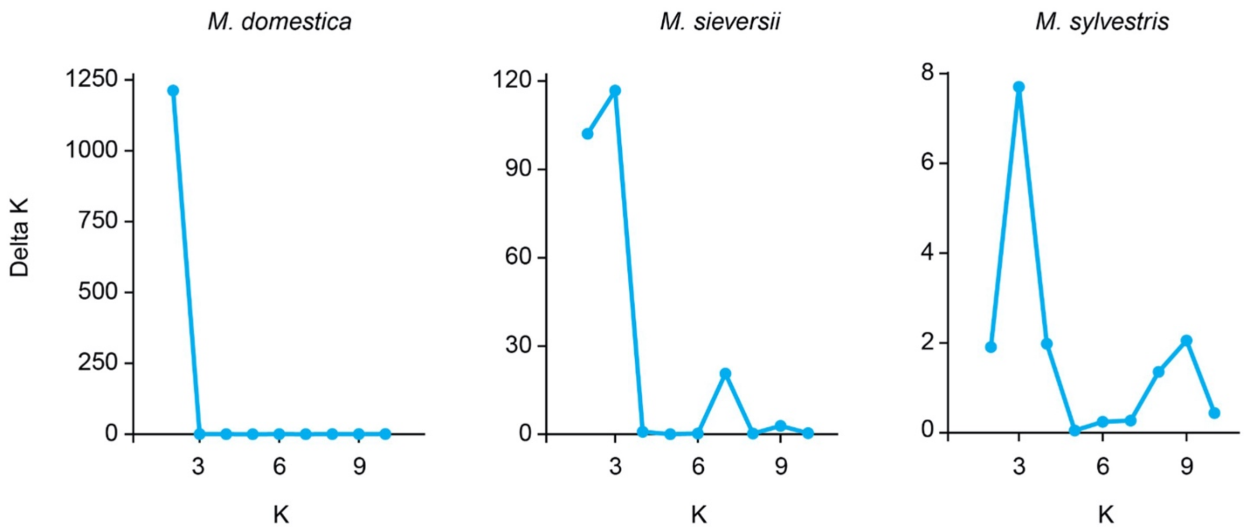

Supplementary Fig. 11 Selection of the optimal number of clusters (*K*) based on the Δ*K* analysis.

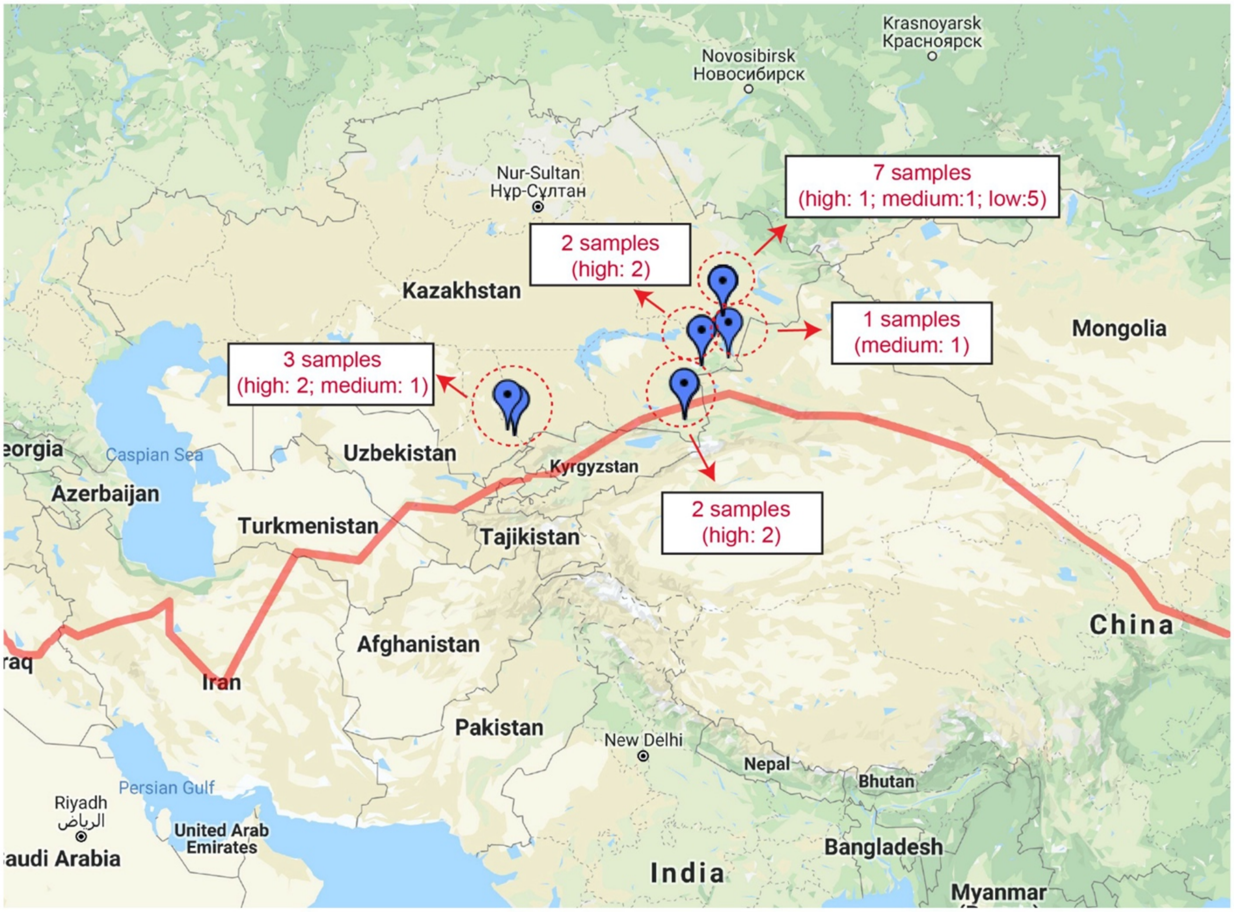

Supplementary Fig. 12 Geographic distribution of *M. sieversii* accessions from Kazakhstan. The geographic information of 15 *M. sieversii* accessions from Kazakhstan were retrieved from the GRIN database (https://www.ars-grin.gov/), and navigated on the Google map. The long red line indicates the route of ancient Silk Road. Samples were classified based on the genome proportion that may have been introgressed.

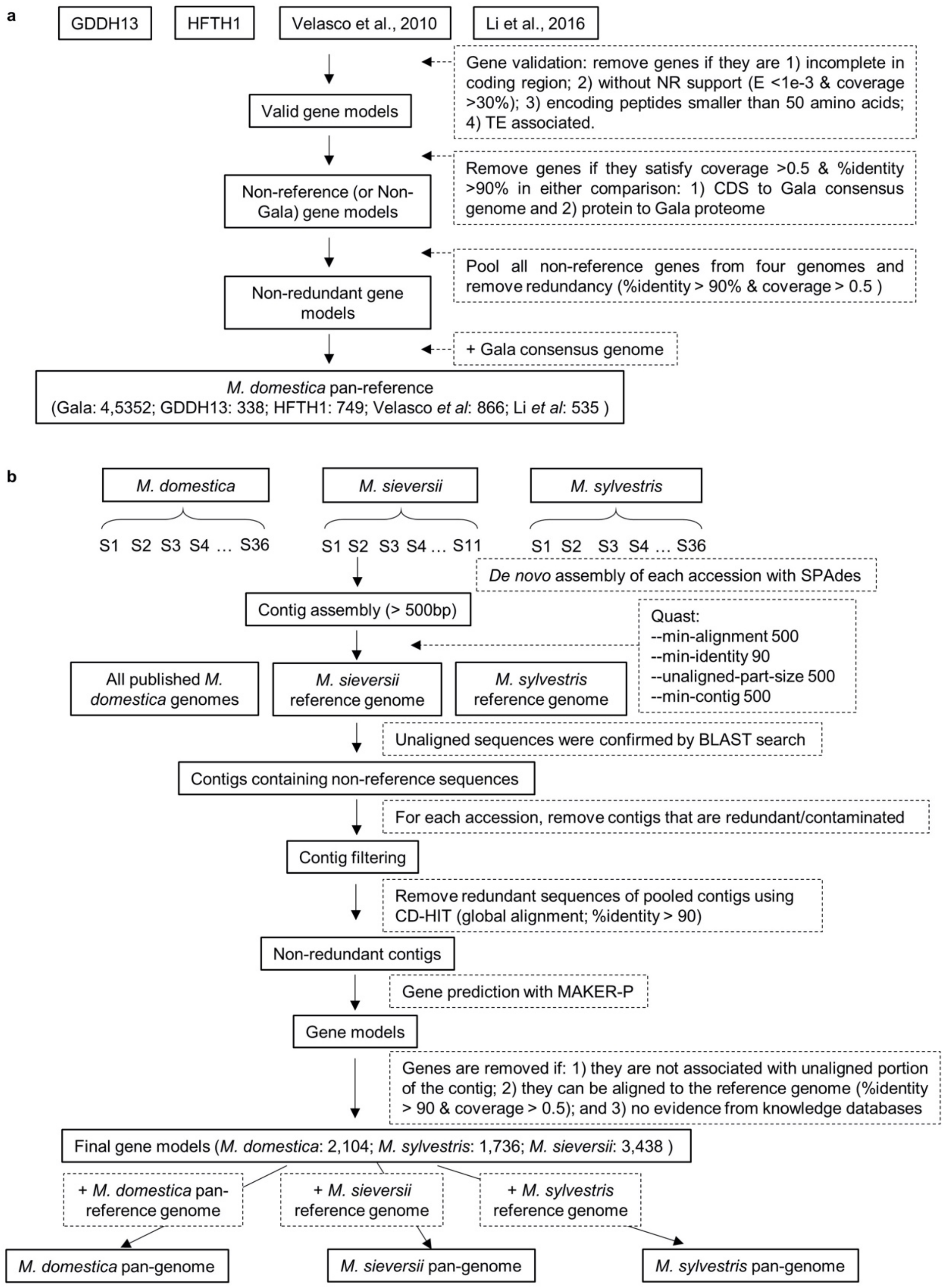

Supplementary Fig. 13 Computational pipeline for apple pan-genome construction. **a**, Strategy for constructing the pan-reference genome of *Malus domestica* from the four published genome assemblies and the Gala consensus assembly. **b**, Strategy for building the pan-genomes of *M. domestica*, *M. sieversii* and *M. sylvestris*.

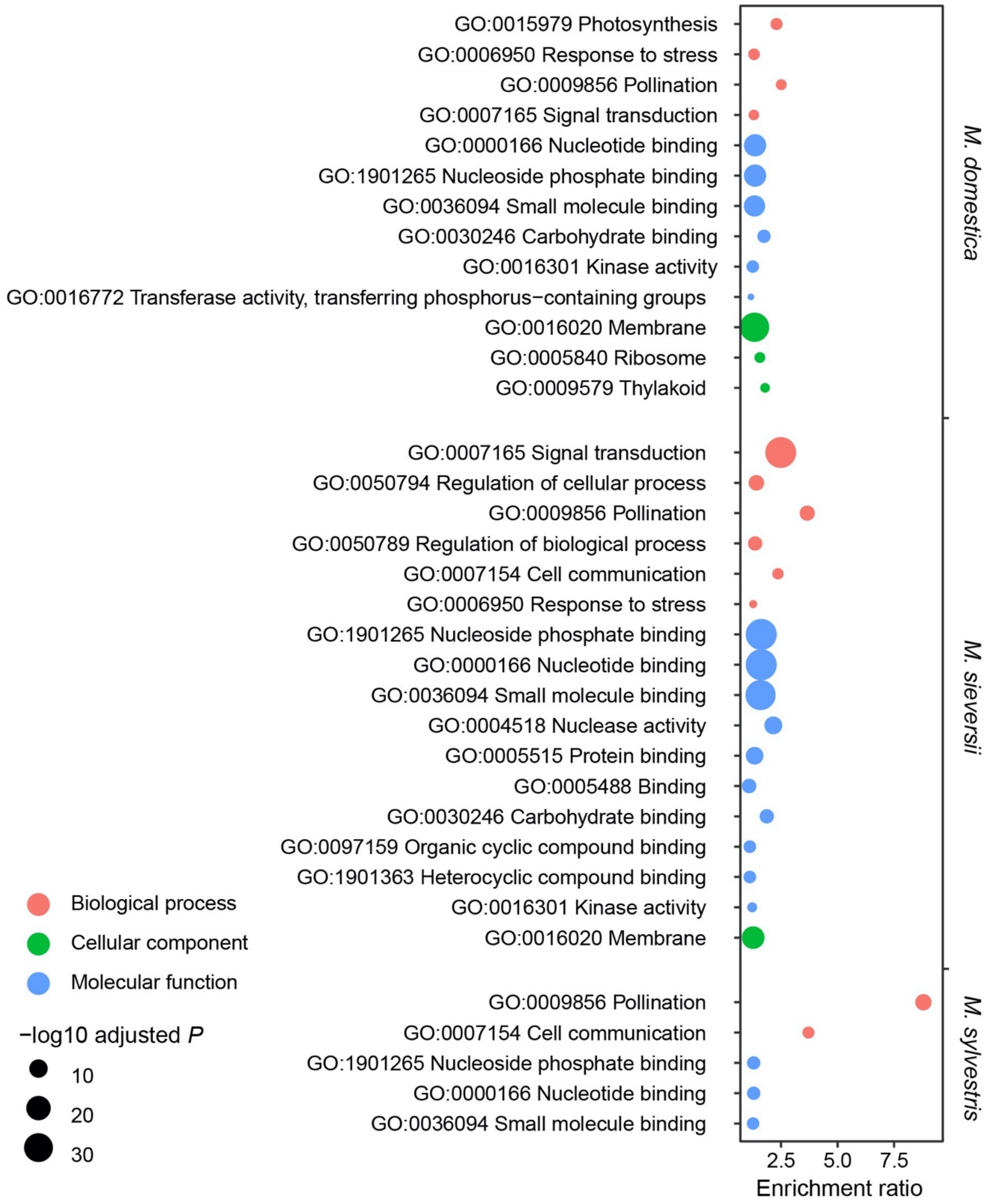

Supplementary Fig. 14 GO term enrichment analysis of novel genes in the pan-genomes of the three *Malus* species. Only GO terms with adjusted *P* value < 0.01 are shown.

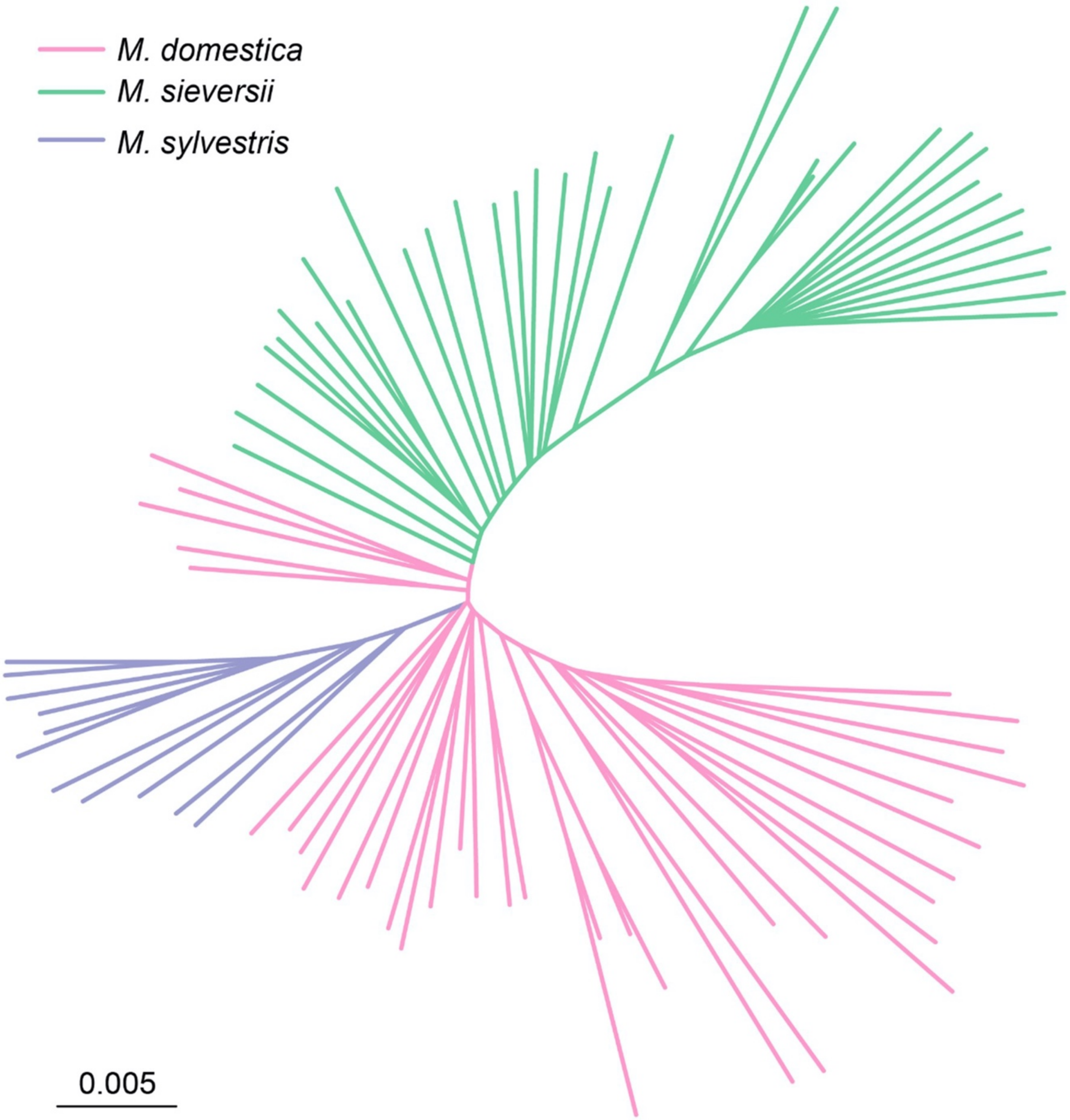

Supplementary Fig. 15 Neighbor-joining phylogeny of the *Malus* accessions constructed using the pan-genome PAVs.

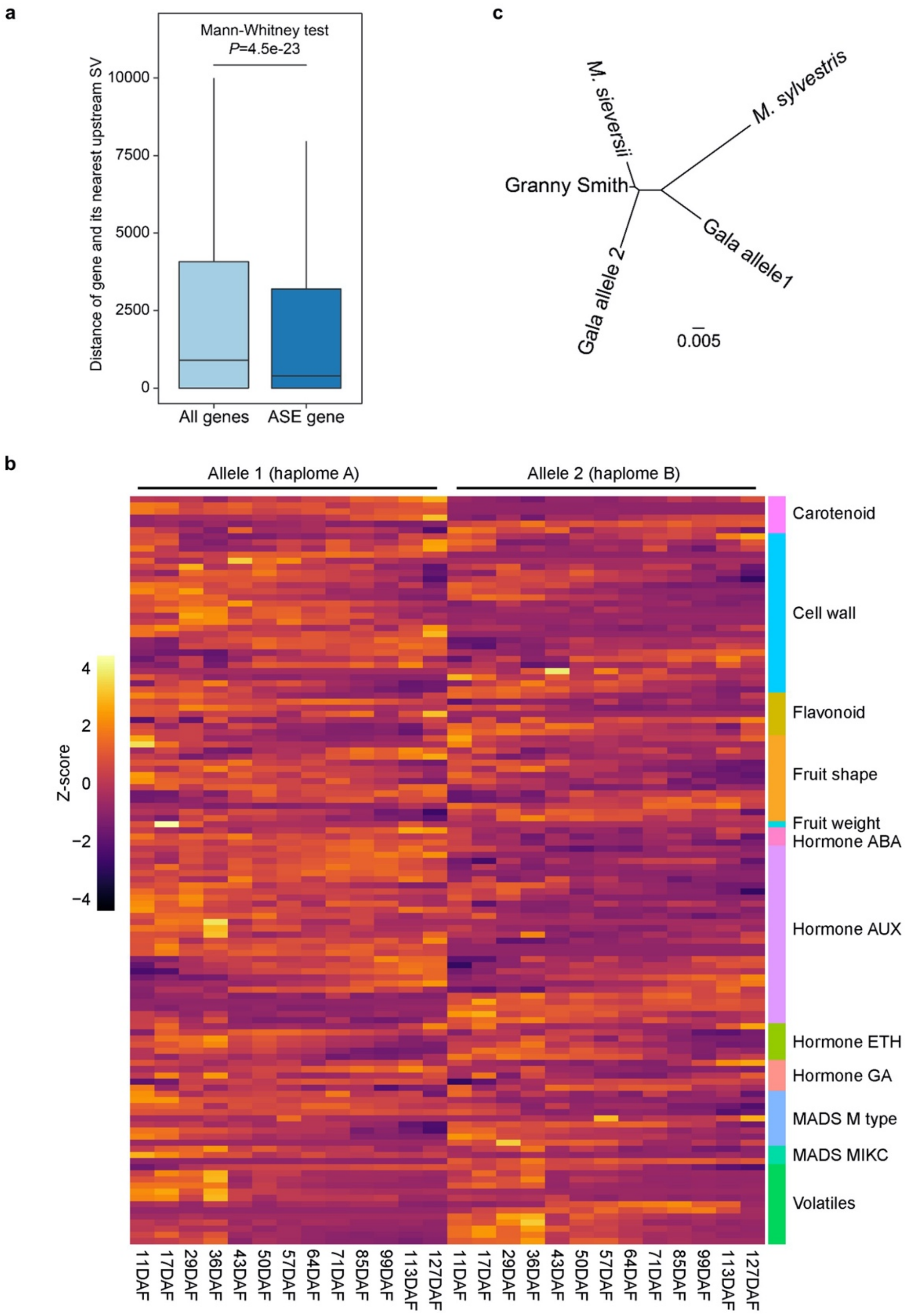

Supplementary Fig. 16. Functional analysis of genes with allele-specific expression. **a**, Box plot showing the distribution of the distance between genes and their upstream nearest SVs. **b**, ASE pattern of genes associated with diverse biological processes. **c**, Unrooted maximum likelihood phylogeny of the *AAT1* gene in *M. sieversii*, *M. sylvestris* and apple cultivars Gala and Granny Smith.
